# Inhibition of GABARAP or GABARAPL1 prevents aminoglycoside- induced hearing loss

**DOI:** 10.1073/pnas.2416453122

**Published:** 2025-02-10

**Authors:** Jinan Li, Seung-Il Oh, Chang Liu, Bo Zhao

**Affiliations:** ^a^Department of Otolaryngology-Head and Neck Surgery, Indiana University School of Medicine, Indianapolis, IN 46202

**Keywords:** hearing loss, hair cell, aminoglycoside, GABARAP, GABARAPL1

## Abstract

Aminoglycosides (AGs) are highly potent, broad-spectrum antibiotics frequently used as first-line treatments for multiple life-threatening infections. Despite their severe ototoxicity, causing irreversible hearing loss in millions of people annually, no preventive therapy has been approved. We previously reported that GABARAP and several other central autophagy proteins are essential for AG-induced hearing loss. This finding opens avenues for the rational design and development of inhibitors that selectively target proteins in this pathway, thereby mitigating AG ototoxicity. In this study, we generated a mouse model with a targeted deletion of GABARAPL1, a homolog of GABARAP, and another model deficient in both GABARAP and GABARAPL1. We found that normal hearing is unaffected by the depletion of these proteins. Remarkably, both proteins are essential for AG-induced hearing loss, with GABARAP playing a more significant role. To further explore the therapeutic potential, we designed and validated short hairpin RNAs targeting the mouse and human *GABARAP* gene. By inhibiting GABARAP expression in inner ear hair cells using adeno-associated virus-mediated RNA interference, we successfully prevented AG-induced hair cell death and subsequent hearing loss. Our findings underscore the critical role of GABARAP in AG ototoxicity and highlight its potential as a therapeutic target for preventing AG-induced hearing loss.

## Significance

Each year, over 20 million cases of hearing loss are estimated to result from aminoglycoside (AG) exposure. Currently, there are no approved therapies to prevent AG-induced hearing loss. Identifying therapeutic targets that can be safely inhibited is, therefore, crucial for preventing AG-induced hearing loss. This study, using genetic approaches along with morphological and functional assays, reveals that both GABARAPL1 and GABARAP are essential for AG-induced hearing loss, with GABARAP playing a more prominent role. Notably, genetic depletion of both proteins in mice does not affect normal hearing, indicating their safety as drug targets. Further functional tests following adeno-associated virus-mediated RNA interference highlight the potential of GABARAP as a therapeutic target to prevent AG-induced hearing loss without compromising normal auditory function.

**Fig. 1.**
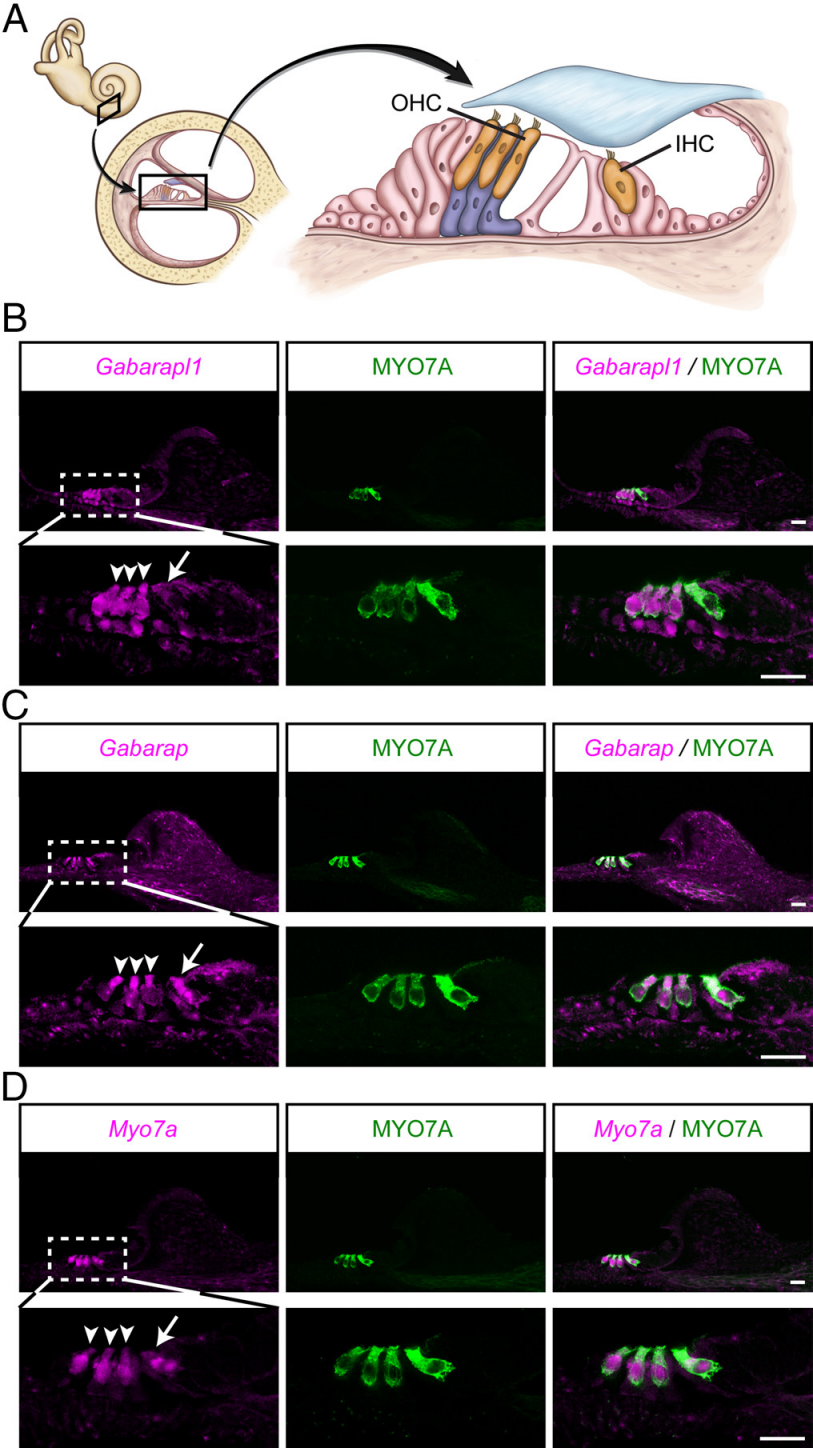
*Gabarapl1* and *Gabarap* are abundantly expressed in cochlear hair cells. (*A*) Schematic illustrating the structure of the inner ear, including a cross-sectional view of the cochlea and the cellular organization within the organ of Corti. Inner hair cell (IHC) and outer hair cells (OHCs) are highlighted in orange, while Deiters’ cells, which support the base of OHCs, are illustrated in lavender blue. (*B*–*D*) RNAscope in situ hybridization analyses were performed to characterize the expression of *Gabarapl1* (*B*), *Gabarap* (*C*), and *Myo7a* (*D*) in P5 wild-type cochlea. Tissue sections were counterstained with MYO7A antibodies to visualize the IHCs (arrows) and OHCs (arrowheads). Note, robust expression of *Gabarapl1* and *Gabarap* in hair cells and some supporting cells, such as Deiters’ cells. (Scale bars, 20 µm.)

Hearing loss is one of the most common health problems, affecting ~1.6 billion people worldwide (1). Aminoglycoside antibiotics (AGs), such as kanamycin (KAN), gentamicin (GEN), amikacin, and tobramycin, are highly potent and broad-spectrum antibiotics frequently used as first-line treatments for multiple life-threatening infections. More than 100 million people worldwide are estimated to be treated with AGs each year (2). However, AGs preferentially kill inner ear hair cells (Fig. 1*A*), the mechanosensory cells that detect sound, and cause permanent hearing loss in 20 to 47% of patients (3–5). It is estimated that more than 20 million new cases per year, accounting for ~60% of all new hearing loss cases, are associated with exposure to AGs (2). Unfortunately, seven decades after the initial reports of severe ototoxic effects of AGs, there is still no approved antidote (6), mainly because the underlying mechanism by which AGs preferentially kill auditory hair cells was unknown making it difficult to identify therapeutic targets.

Over the years, substantial insights have been obtained regarding the routes through which AGs enter hair cells. AGs that are administered systemically are trafficked across the blood-endolymph barrier and preferentially enter hair cells across their apical membranes (7). The mechanotransduction (MET) channels, which convert sound-induced vibrations into electrical signals and are localized near the tips of stereocilia that protrude from the apical surface of hair cells, are the major entry routes into hair cells for AGs (8–11). Genetically mutated mice, which exhibit impaired MET and therefore profound hearing loss, are resistant to AG-induced hair cell death due to the inability of their hair cells to take up AGs (9, 12–14). In addition to the MET channels, studies have also found that increased expression of transient receptor potential vanilloid-1 during inflammation facilitates the uptake of AGs by hair cells (15). However, once AGs enter hair cells, the mechanisms by which they cause hair cell death were not well understood.

We previously found that upon entering hair cells, AGs bind to and trigger rapid translocation of RIPOR2, a protein that is abundantly expressed in cochlear hair cells and essential for normal hearing (13, 16–18). Translocated RIPOR2 aberrantly activates the autophagy pathway, by interacting with GABARAP, a member of the autophagy-related protein 8 (Atg8) family (13, 19). Genetic elimination or reduction in GABARAP expression completely prevents AG-induced hair cell death and subsequent hearing loss, without affecting hair cell development or normal hearing, indicating that GABARAP is a potential therapeutic target for mitigating the ototoxic side effects of AGs (13). Notably, GABARAPL1, the close homolog sharing 87% amino acid identity with GABARAP, is also abundantly expressed in cochlear hair cells (20, 21) and interacts with RIPOR2 (13). Given that genetic compensation in response to gene deletion is a common phenomenon (22), the extent to which GABARAPL1 compensates for GABARAP’s function, if any, in hair cell development and auditory function in *Gabarap*-null mutant mice remains uncertain. Determining the extent to which GABARAP and GABARAPL1 are essential for normal hearing, and whether inhibiting GABARAP—or GABARAPL1, if more effective—can mitigate AG-induced hearing loss without compromising normal hearing, will be of significant importance.

To date, all previously reported genetic mouse models that are entirely resistant to AG ototoxicity are null mutants (13). These mutants, such as *Gabarap-*knockout mice (13) have the target gene deleted in all tissues. It remains unclear whether the resistance to ototoxicity from systemically administered AG results from a deficiency in the expression of the corresponding gene in the inner ear, or in some other tissues, such as the liver, which facilitates the removal of toxic substances from the body through the detoxification process (23). Determining the tissue where the targeted gene should be inhibited to mitigate AG-induced hearing loss will provide crucial insights for selecting future drug administration strategies. Thus, to address this question, we aimed to specifically reduce the expression of *Gabarap* in the inner ear. Recombinant adeno-associated virus (AAV), considered one of the most promising in vivo gene delivery tools, is able to deliver genetic materials to a specific organ via local injection (24). Notably, AAV-based gene therapy has been demonstrated to be effective and safe in numerous preclinical studies and clinical trials (24) and has been shown to improve auditory function in mouse models of hereditary hearing loss and in human clinical trials over the past few years (25–27). Using AAV-based RNA interference (RNAi), we were able to precisely reduce the expression of *Gabarap* in the inner ear and investigate its efficacy in preventing AG-induced ototoxicity.

In the present study, we generated a *Gabarapl1* single knockout mouse line, and a *Gabarap*/*Gabarapl1* double-knockout mouse line. We found that normal hearing is not affected by the depletion of *Gabarap* and/or *Gabarapl1* expression. Remarkably, genetic knockout of *Gabarap* prevents AG-induced hearing loss entirely, whereas knockout of *Gabarapl1* prevents AG-induced hearing loss only partially. This finding suggests that *Gabarap* is the dominant homolog essential for AG-induced hearing loss. Next, we designed and validated short hairpin RNAs (shRNAs) that specifically target *Gabarap* of both mouse and human origin. Then, AAV expressing shRNA was administered into the inner ear via local injection. Our findings showed that the targeted inhibition of *Gabarap* expression in the inner ear effectively prevents hair cell death and subsequent hearing loss caused by systemic AG administration, suggesting that *Gabarap* is a promising therapeutic target for mitigating the ototoxicity of AGs.

## Results

### Generation of *Gabarapl1*-Null Mutant Mouse.

GABARAP and GABARAPL1 share 87% amino acid identity (*SI Appendix*, Fig. S1*A*) and function redundantly in the autophagy pathway (28). According to published RNA-Seq studies, *Gabarap* and *Gabarapl1* are highly expressed in hair cells (20, 21). To extensively study the expression profiles of *Gabarap* and *Gabarapl1* in the inner ear, specific RNAscope in situ hybridization probes targeting each gene were designed. Indeed, RNAscope revealed a robust expression of both *Gabarap* and *Gabarapl1* in cochlear OHCs and IHCs, which are characterized by abundant Myosin 7A (MYO7A) expression (Fig. 1 *A*–*D* and *SI Appendix*, Fig. S1*B*). Both genes were also expressed in some surrounding cells, such as Deiters' cells (Fig. 1 *A*–*C*), which is consistent with previous single-cell RNA-seq studies (29). As a control, the RNAscope probe targeting *Myo7a* produced a strong signal in both OHCs and IHCs, overlapping with the immunostaining signal from the MYO7A antibody (Fig. 1*D*). In line with RNA-Seq results (20, 21), which indicate higher expression levels of *Gabarap* compared with *Gabarapl1* in hair cells, qRT-PCR analysis also suggested that *Gabarap* is expressed at higher levels than *Gabarapl1* in the inner ear (*SI Appendix*, Fig. S1*C*).

**Fig. 2.**
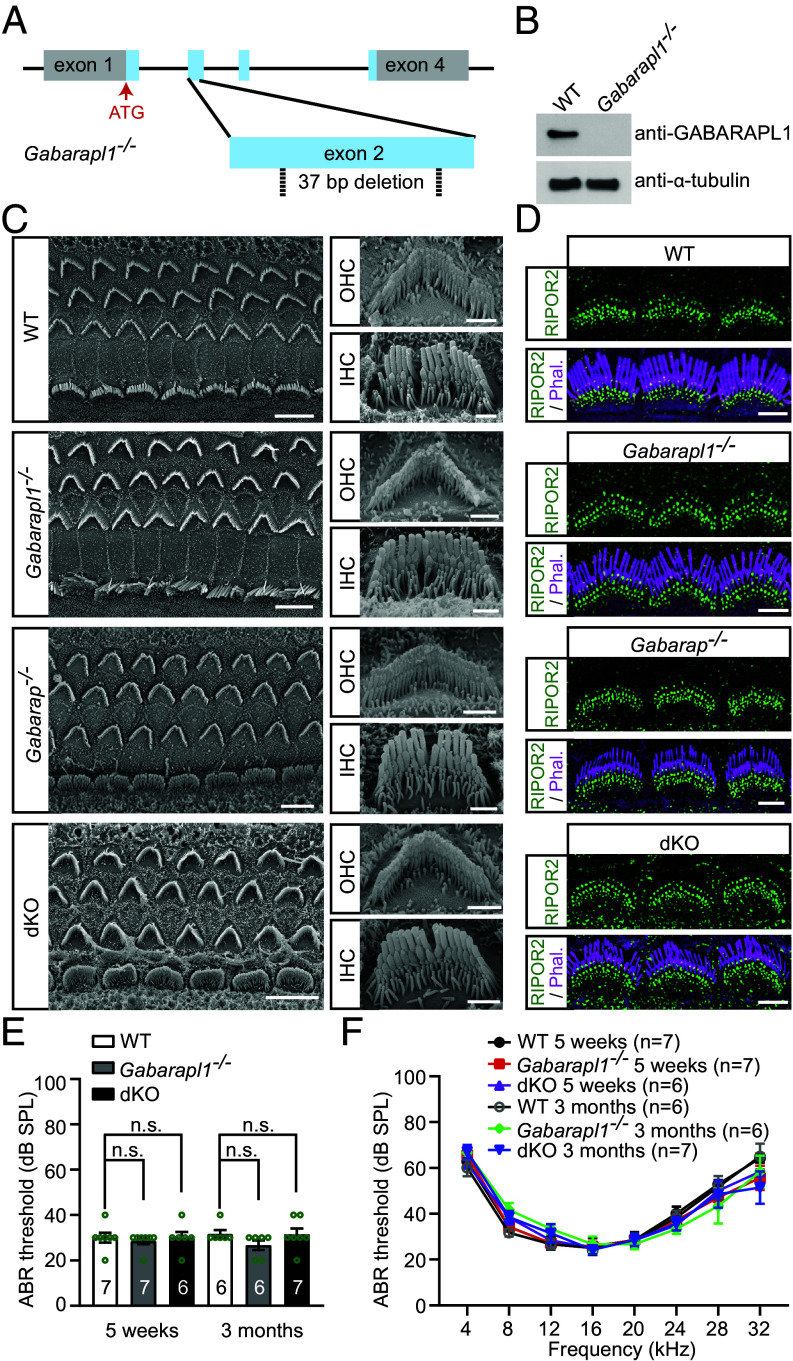
GABARAP and GABARAPL1 are not required for stereocilia morphogenesis or for normal hearing. (*A*) Diagram of the strategy to generate *Gabarapl1*-deficient mice. Two sgRNAs targeting the exon 2 of *Gabarapl1* induced a 37-bp nucleotide deletion. (*B*) Western blotting was used to analyze P7 wild-type and *Gabarapl1^−/−^* cochlear samples. Note, the ~15 kDa specific band was detected in wild-type but not in the *Gabarapl1^−/−^* inner ear samples. α-tubulin was used as the loading control. (*C*) Scanning electron microscopy images showing P7 wild-type, *Gabarapl1^−/−^*, *Gabarap^−/−^*, and *Gabarap^−/−^Gabarapl1^−/−^* (dKO) cochlear epithelia. In each group, more than three mice were used. (*D*) P7 wild-type, *Gabarapl1^−/−^*, *Gabarap^−/−^*, and *Gabarap^−/−^Gabarapl1^−/−^* (dKO) cochlear whole mounts were stained for RIPOR2 (green) and phalloidin (magenta) to reveal stereocilia. In each group, more than three mice were used. (*E* and *F*) ABR thresholds for click stimuli (*E*) and pure tones (*F*) in wild-type, *Gabarapl1^−/−^*, and *Gabarap^−/−^Gabarapl1^−/−^* (dKO) mice at 5 wk and 3 mo of age. Mouse numbers are indicated in the figure. Data are represented as the mean ± SEM. n.s., not significant by Student’s *t* test (*E*). No significant difference by two-way ANOVA was detected between wild-type and *Gabarapl1^−/−^* mice, or between wild-type and *Gabarap^−/−^Gabarapl1^−/−^* (dKO) mice (*F*). [Scale bars, (*C*) *Left* panel, 5 µm; *Right* panel, 1 µm; (*D*) 5 µm.]

To gain insight into the functions of GABARAPL1 in hair cells, we introduced a 37-bp nucleotide deletion in the exon 2 of *Gabarapl1* using the CRISPR/Cas9 system. The frameshift caused by the deletion in the *Gabarapl1* genomic locus resulted in a change of amino acid after residue 40, followed by an early stop codon (Fig. 2*A* and *SI Appendix*, Fig. S1 *D*–*G*). To confirm whether this mouse is a *Gabarapl1*-null mutant, cochlear tissue was dissected from pups at postnatal day 7 (P7), and western blotting was performed. Antibodies specific to GABARAPL1, which do not recognize GABARAP or GATE-16/GABARAPL2—another homolog sharing 71% amino acid identity with GABARAPL1 and 58% amino acid identity with GABARAP (*SI Appendix*, Fig. S1 *A* and *H*), detected a ~15 kDa protein in wild-type mice but not in the *Gabarapl1*-mutant mice (Fig. 2*B*). This result suggests that this mouse is a *Gabarapl1*-null mutant. Hereafter, we will refer to this mouse as *Gabarapl1^−/−^* mouse.

### Neither GABARAP Nor GABARAPL1 Is Required for Stereocilia Morphogenesis or for Normal Hearing.

As previously determined by coimmunoprecipitation assays, both GABARAP and GABARAPL1 bind strongly with RIPOR2 (13), a protein that is required for stereocilia morphogenesis and AG-induced hearing loss (13, 16–18). Mice lacking RIPOR2 have disorganized stereocilia and profound early-onset hearing loss (16–18). To investigate the extent to which GABARAPL1 is required for the morphogenesis of stereocilia, scanning electron microscopy and whole mount immunostaining were performed. Hair bundle development and RIPOR2 localization in hair cells were minimally affected at P7 in mice lacking GABARAP or GABARAPL1 (Fig. 2 *C* and *D*). Given the redundant functions of GABARAP and GABARAPL1 in the autophagy pathway, we asked whether GABARAP and GABARAPL1 compensate for one another’s function in the single null mutant mouse. Thus, we generated *Gabarap* and *Gabarapl1* double-knockout mice (referred to as *Gabarap^−/−^Gabarapl1^−/−^*) by crossing *Gabarap^−/−^* mice with *Gabarapl1^−/−^* mice. The hair bundle morphology of *Gabarap^−/−^Gabarapl1^−/−^* mice was then characterized. Scanning electron microscopy and whole mount immunostaining revealed that stereocilia morphology and RIPOR2 localization were unaffected in the double-knockout mice (Fig. 2 *C* and *D*). Next, the styryl dye FM1-43 was used to assess MET activity in hair cells. Consistent with previous studies (12, 30), rapid FM1-43 uptake was abolished in hair cells lacking MET, either through genetic knockout of TMIE, an essential subunit of MET channel (31, 32), or via pharmacological treatment with 5 mM EGTA (33). Remarkably, robust intracellular fluorescence following a brief 30-s exposure to 5 µM FM1-43 was observed in *Gabarapl1^−/−^* and *Gabarap^−/−^Gabarapl1^−/−^* hair cells (*SI Appendix*, Fig. S2 *A* and *B*), indicating the presence of functional MET in these mutant mice. Next, to characterize the auditory function of these mice, the auditory brainstem response (ABR) to broadband click stimuli was measured. Both *Gabarapl1^−/−^* single-knockout mice and *Gabarap^−/−^Gabarapl1^−/−^*double-knockout mice had normal hearing at 6 wk and 3 mo of age (Fig. 2*E*). Furthermore, pure tone audiometry revealed that hearing thresholds were unaffected across the entire tested frequency spectrum (4 to 32 kHz) (Fig. 2*F*). These results suggest that GABARAP and GABARAPL1 are dispensable for normal hearing.

### GABARAPL1 and GABARAP Colocalize with RIPOR2 in Hair Cells Following AG Exposure.

**Fig. 3.**
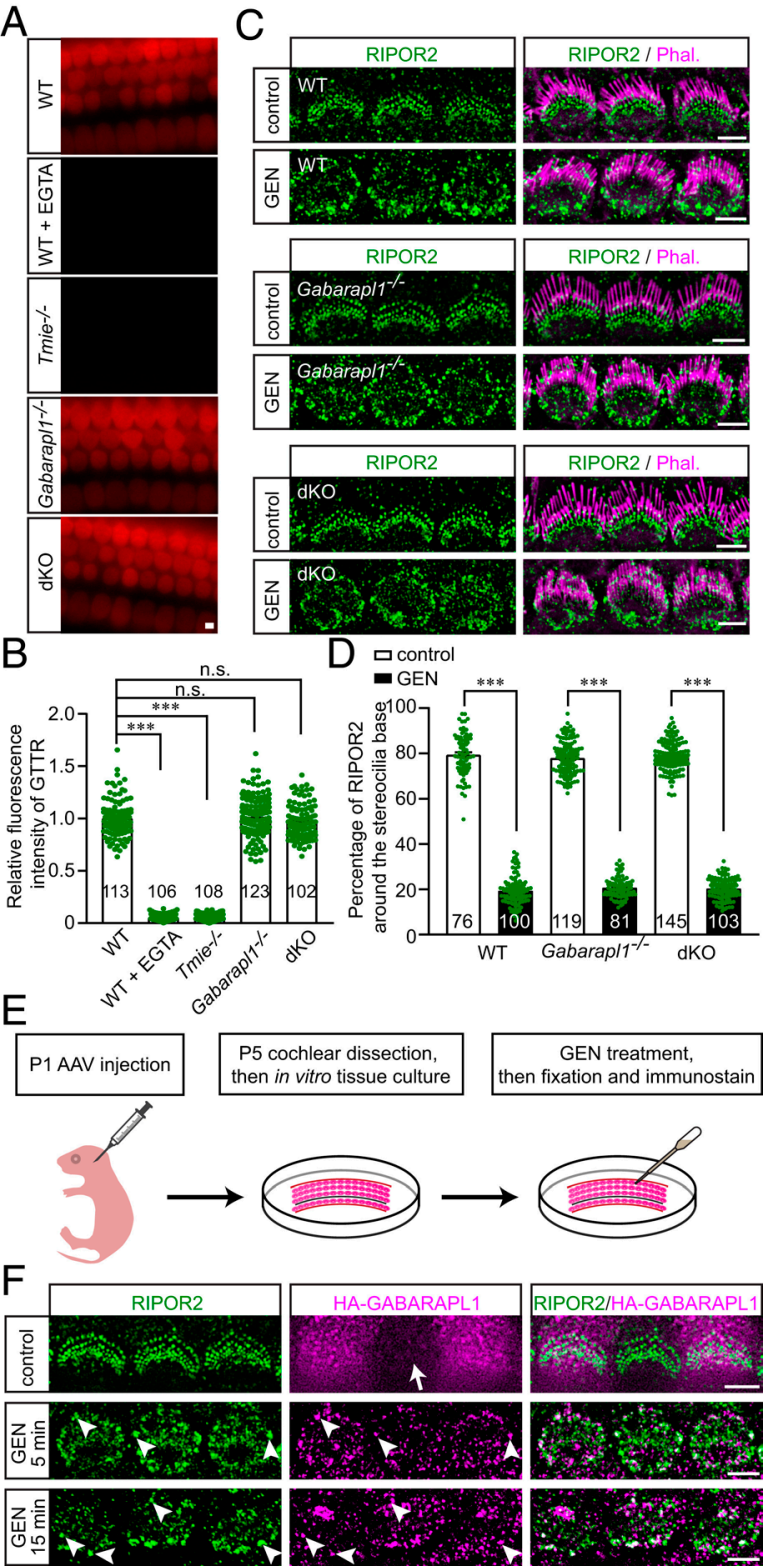
GABARAPL1 colocalizes with RIPOR2 in hair cells following AG exposure. (*A*) P4 wild-type, *Gabarapl1^−/−^*, *Gabarap^−/−^Gabarapl1^−/−^* (dKO), and *Tmie^−/−^* cochlear explants were treated with gentamicin-conjugated Texas Red (GTTR) for 1 min. Note, robust uptake of GTTR in wild-type, *Gabarapl1^−/−^*, and dKO hair cells. GTTR uptake was significantly reduced in wild-type hair cells after 30 min of pretreatment with 5 mM EGTA, as well as in *Tmie^−/−^* hair cells. (*B*) Quantification results of the GTTR fluorescence intensity as shown in (*A*). In each group, three mice were used, and more than 30 hair cells per mouse were analyzed. The total number of cells analyzed per group is indicated in the figure. Data are represented as the mean ± SEM. n.s., not significant, ***P < 0.001 by Student’s *t* test. (*C*) P4 wild-type, *Gabarapl1^−/−^*, and *Gabarap^−/−^Gabarapl1^−/−^* (dKO) cochlear explants were treated with 1 mM GEN for 15 min, fixed, and then stained for RIPOR2 (green) and phalloidin (magenta). Note, robust translocation of RIPOR2 from base of stereocilia to the pericuticular area in the hair cells. (*D*) Percentage of RIPOR2 around the stereocilia base as shown in (*C*) was measured and quantified. In each group, more than three mice were used, and more than 20 hair cells per mouse were analyzed. The total number of cells analyzed per group is indicated in the figure. Data are represented as the mean ± SEM. n.s., not significant, ***P < 0.001 by Student’s *t* test. (*E*) Experimental paradigm of determining the localization of GABARAPL1 in hair cells. AAVs expressing HA-tagged GABARAPL1, under the control of a CBh promoter, were injected into the wild-type inner ear via the posterior semicircular canal. Cochlear explants were then dissected and exposed to GEN. Then tissues were fixed for immunostaining. (*F*) Cochlear explants were stained for RIPOR2 (green) and HA (magenta). Note, colocalization of RIPOR2 and HA-GABARAPL1 (arrowheads) at the pericuticular area after GEN treatment. A non-AAV-infected cell (arrow) in the middle of the top image served as the control for antibody specificity. (Scale bars, 5 µm.)

AGs enter hair cells mainly through the MET channel (8–11), which is formed by TMC1/TMC2, TMIE, and other components (12, 30–32, 34–36). Genetic deletion of TMC1/TMC2 or TMIE results in the loss of robust AG uptake in hair cells (12, 13) (Fig. 3*A*). To investigate whether GABARAP and GABARAPL1 are required for AG uptake by hair cells, cochlear explants were dissected from P4 *Gabarapl1^−/−^* and *Gabarap^−/−^Gabarapl1^−/−^* mice, and subsequently incubated with GEN-conjugated Texas Red (GTTR) for 1 min. The robust uptake of GTTR in *Gabarapl1^−/−^* and *Gabarap^−/−^Gabarapl1^−/−^* hair cells was not significantly different from that in wild-type hair cells (Fig. 3 *A* and *B*), suggesting that depletion of GABARAP and GABARAPL1 expression has no effect on AG uptake by hair cells. In line with previous studies (9, 11, 12), robust GTTR uptake was abolished in hair cells lacking MET subunit TMIE (31, 32), as well as in wild-type hair cells with impaired MET following EGTA treatment (33) (Fig. 3 *A* and *B*).

Our previous studies revealed that AGs trigger a rapid translocation of RIPOR2 from the base of stereocilia to the pericuticular area within minutes (13). GABARAP and GABARAPL1, in addition to their roles as essential components in the autophagy pathway, are known for their involvement in regulating the transport and cell surface expression of various receptors and ion channels in different types of cells (37–42). Thus, the GEN-triggered translocation of RIPOR2 was characterized in *Gabarapl1^−/−^* single-knockout mice. Cochlear explants were dissected from P4 mice, treated with 1 mM GEN for 15 min, and then fixed for immunostaining to characterize RIPOR2 localization. A rapid translocation of RIPOR2 from the stereocilia base to the pericuticular region was induced by GEN in *Gabarapl1^−/−^* hair cells, without any significant difference when compared with wild-type hair cells (Fig. 3 *C* and *D*). It is possible that GABARAP may compensate for the function of GABARAPL1 in *Gabarapl1^−/−^* mice. Thus, RIPOR2 translocation was characterized in *Gabarap^−/−^Gabarapl1^−/−^* double-knockout hair cells. Similar to that in wild-type hair cells, GEN triggered a robust translocation of RIPOR2 in *Gabarap^−/−^Gabarapl1^−/−^* double-knockout hair cells (Fig. 3 *C* and *D*).

To investigate whether GABARAPL1 is involved in AG ototoxicity, its localization in wild-type hair cells treated with or without AG was studied. Due to a lack of specific antibodies for immunostaining, we packaged an AAV vector containing the CBh promoter to drive HA-tagged GABARAPL1 expression (*SI Appendix*, Fig. S3). AAV was injected into the inner ear of P1 wild-type mice through the posterior semicircular canal. Cochlear explants were then dissected at P5 and treated with GEN (Fig. 3*E*). Similar to GABARAP (*SI Appendix*, Fig. S4*A*), without GEN treatment, HA-GABARAPL1 diffused in hair cells. Notably, after 5- or 15-min of GEN treatment, HA-GABARAPL1 was concentrated in the pericuticular area and colocalized with RIPOR2 (Fig. 3*F*), indicating that GABARAPL1 is involved in the RIPOR2-mediated AG-ototoxicity pathway. Similar to GABARAP (*SI Appendix*, Fig. S4*B*), HA-GABARAPL1 did not significantly accumulate in the pericuticular area of *Ripor2^+/−^* heterozygous hair cells with reduced RIPOR2 expression following GEN treatment (*SI Appendix*, Fig. S4*C*). This suggests that RIPOR2 regulates GABARAPL1 translocation and functions upstream of GABARAPL1.

### GABARAPL1 and GABARAP Are Essential for AG-Induced Hair Cell Death and Subsequent Hearing Loss.

**Fig. 4.**
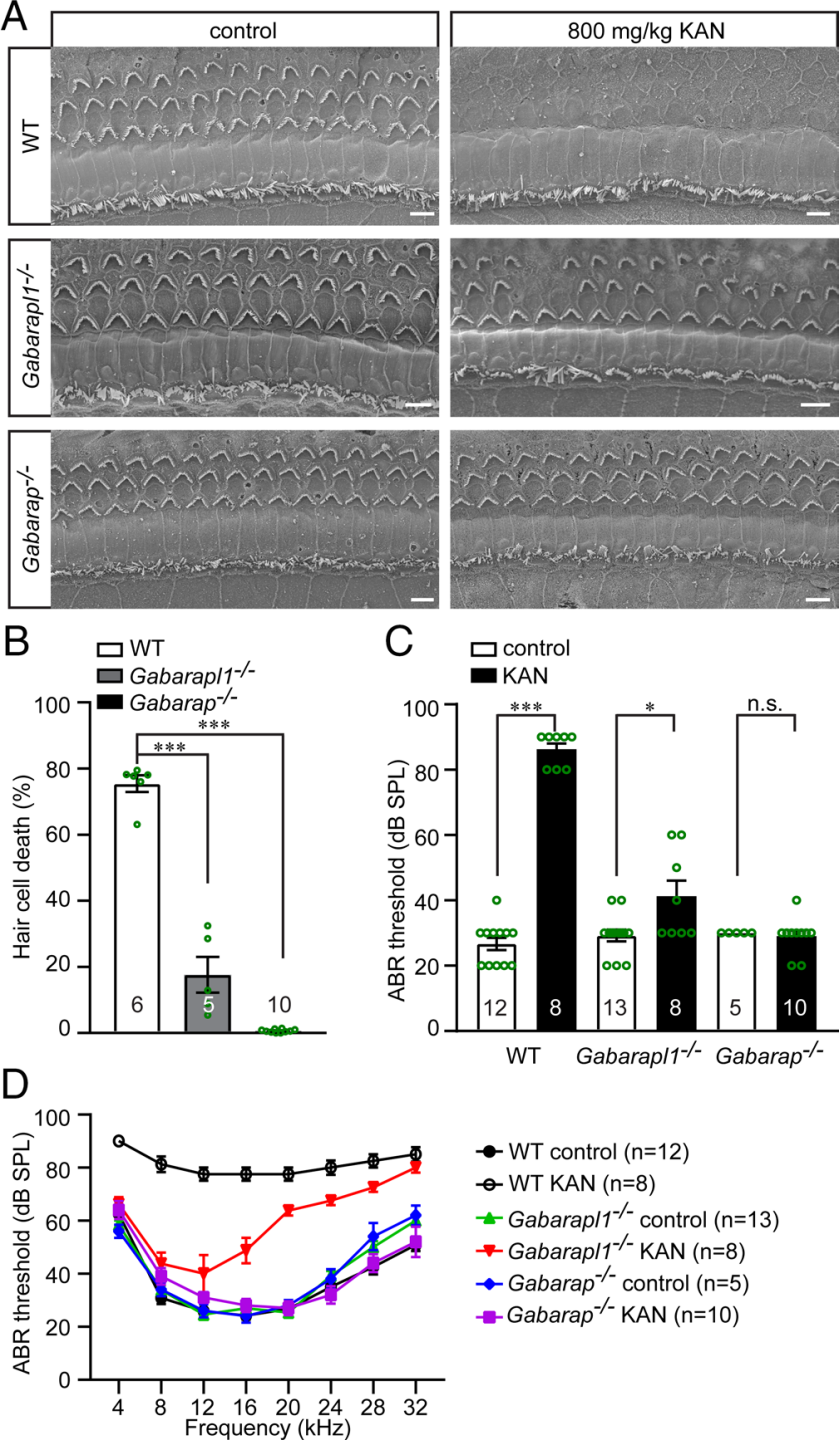
GABARAPL1 and GABARAP are essential for AG-induced hair cell death and subsequent hearing loss. (*A*) 3-wk-old wild-type, *Gabarapl1^−/−^*, and *Gabarap^−/−^* mice were treated with 800 mg/kg kanamycin (KAN) for 14 consecutive days. Two to four days after the final KAN injection, cochleae were then dissected and fixed for scanning electron microscopy. Middle regions of the cochlea were shown. (*B*) Percentages of hair cell death in wild-type, *Gabarapl1^−/−^*, and *Gabarap^−/−^* mice were quantified. The numbers of analyzed mice are indicated in the figure. Data are represented as the mean ± SEM. ***P < 0.001 by Student’s *t* test. (*C*) ABR thresholds for click stimuli in wild-type, *Gabarapl1^−/−^*, and *Gabarap^−/−^* mice, measured 2 to 4 d after the final KAN injection. The numbers of analyzed mice are indicated. Data are represented as the mean ± SEM. n.s., not significant, **P < 0.01, ***P < 0.001 by Student’s *t* test. (*D*) ABR thresholds for pure tones in wild-type, *Gabarapl1^−/−^*, and *Gabarap^−/−^* mice, measured 2 to 4 d after the final KAN injection. Data are represented as the mean ± SEM. *P* < 0.001 between nontreated and KAN-treated wild-type mice, *P* < 0.001 between nontreated and KAN-treated *Gabarapl1^−/−^* mice, no significant difference between nontreated and KAN-treated *Gabarap^−/−^* mice (two-way ANOVA). (Scale bars, 5 µm.)

Next, the extent to which GABARAPL1 is required for AG ototoxicity was determined using a well-established method for studying AG ototoxicity: subcutaneous injection of 800 mg/kg KAN twice daily for 14 consecutive days, which induces robust hair cell death and profound hearing loss across all frequencies in wild-type C57BL/6J mice (6, 13). After 14 d of KAN treatment, cochleae from *Gabarapl1^−/−^* mice, *Gabarap^−/−^* mice, and wild-type C57BL/6J control mice were dissected, and scanning electron microscopy was performed. In wild-type mice, only ~18% of hair cells survived after 800 mg/kg KAN treatment. In *Gabarapl1^−/−^* mice treated with KAN, more than 80% of the hair cells survived (Fig. 4 *A* and *B*). Remarkably, minimal hair cell loss was detected in *Gabarap^−/−^* mice after KAN treatment (Fig. 4 *A* and *B*). These results suggest that ablating GABARAPL1 provides partial protection against AG-induced hair cell death, whereas ablating GABARAP offers complete protection against hair cell death induced by AG.

Consistent with the histological results, wild-type mice were profoundly deaf across the entire tested frequency spectrum (4 to 32 kHz) after 800 mg/kg KAN treatment, as determined by click ABR and pure-tone audiometry (Fig. 4 *C* and *D*). In *Gabarapl1^−/−^* mice, the same KAN treatment resulted in a ~16 dB hearing threshold elevation, as determined by click ABR; accompanied by mild hearing loss at lower frequencies and moderate-to-severe hearing loss at higher frequencies, as determined by pure-tone audiometry (Fig. 4 *C* and *D*). Notably, no significant hearing threshold elevation across the entire tested frequency spectrum was detected in *Gabarap^−/−^* mice following KAN treatment (Fig. 4 *C* and *D* and *SI Appendix*, Fig. S5). These results suggest that both GABARAP and GABARAPL1 are essential for AG-induced hearing loss and that GABARAP has a more prominent function than GABARAPL1.

### Validation of shRNAs that Specifically Target GABARAP.

Given that GABARAP, compared to GABARAPL1, plays a dominant role in AG ototoxicity, and that genetic ablation of GABARAP does not affect normal hearing, we aim to develop drug modalities to inhibit GABARAP expression, thereby mitigating AG-induced hearing loss. Additionally, to date, all genetic mouse models that have been reported to be entirely resistant to AG ototoxicity are null mutants (13). Thus, we asked whether reducing the expression of *Gabarap* specifically in the inner ear could prevent hearing loss induced by AG that is administered systemically. Thus, we aimed to use AAV-based RNAi to reduce *Gabarap* expression, specifically in the inner ear.

**Fig. 5.**
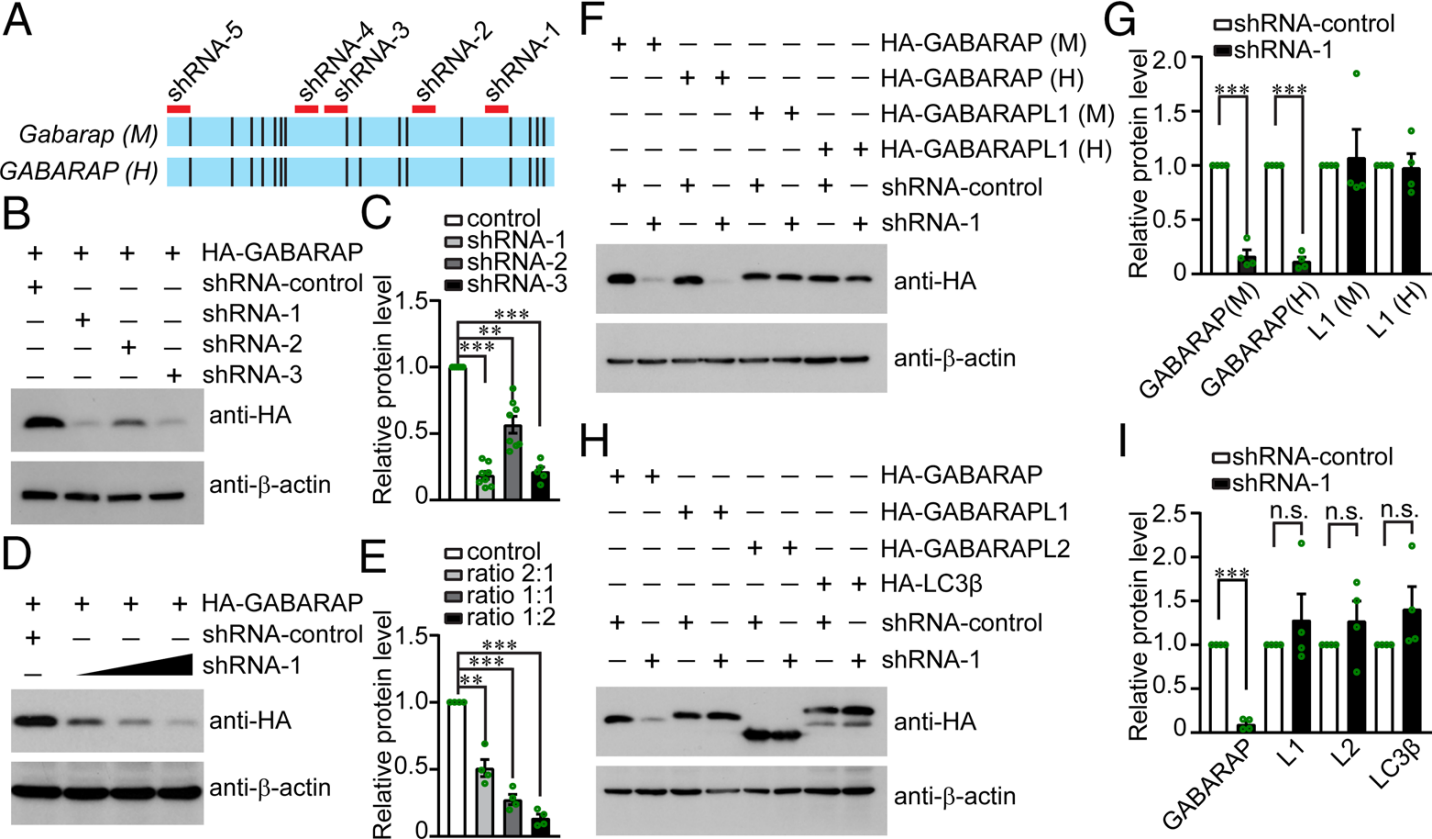
Validation of shRNAs that specifically target *Gabarap*. (*A*) The alignment of the coding sequences of the mouse and human *GABARAP* genes, represented as *Gabarap* (M) and *GABARAP* (H) in the figure respectively. Identical nucleotides are colored cyan, whereas mismatched nucleotides are colored black. Five shRNAs (red bars above) that target identical sequences were designed. (*B*) A plasmid expressing HA-tagged mouse GABARAP, along with a plasmid expressing shRNA, were transfected into HEK293 cells in an equimolar ratio. Western blotting was carried out to evaluate shRNA knockdown efficiency. Scrambled shRNA (shRNA-control) was used as the control, and β-actin was used as the loading control. (*C*) Relative expression level of HA-GABARAP as shown in (*B*). All values were normalized to β-actin and are shown as relative to samples treated with shRNA-control. Data are represented as the mean ± SEM. **P < 0.01, ***P < 0.001 by Student’s *t* test. (*D*) shRNA-1 knocked down mouse GABARAP efficiently in a dose-dependent manner. Different ratios (2:1; 1:1, and 1:2) of the plasmid expressing HA-GABARAP and plasmid expressing shRNA-1 were used. Scrambled shRNA was used as the shRNA control, and β-actin was used as the loading control. (*E*) Quantification of the HA-GABARAP expression as shown in (*D*). All values were normalized to β-actin and are shown as relative to samples treated with shRNA-control. Data are represented as the mean ± SEM. **P < 0.01, ***P < 0.001 by Student’s *t* test. (*F*) Plasmid expressing mouse GABARAP, human GABARAP, mouse GABARAPL1, or human GABARAPL1, along with either the shRNA-1 or shRNA-control plasmid, were transfected into HEK293 cells in an equimolar ratio. shRNA-1 efficiently knocks down both mouse and human GABARAP. shRNA-1 did not affect the expression of mouse or human GABARAPL1. Scrambled shRNA was used as the shRNA control, and β-actin was used as the loading control. (*G*) Quantification of the HA-GABARAP expression as shown in (*F*). Data are represented as the mean ± SEM. ***P < 0.001 by Student’s *t* test. (*H*) Plasmid expression mouse GABARAP, GABARAPL1, GATE-16/GABARAPL2, or LC3β, along with the plasmid expressing shRNA-1, were transfected into HEK293 cells in an equimolar ratio. shRNA-1 has no effect on the expression of GABARAPL1, GATE-16/GABARAPL2, or LC3β. Scrambled shRNA was used as the shRNA control, and β-actin was used as the loading control. (*I*) Quantification of the expression of GABARAP, GABARAPL1, GATE-16/GABARAPL2, and LC3β as shown in (*H*). Data are represented as the mean ± SEM. n.s., not significant, ***P < 0.001 by Student’s *t* test.

*Gabarap* is highly conserved across species. The coding sequences of mouse and human *Gabarap* mRNAs exhibit ~95% nucleotide identity (Fig. 5*A*). With future clinical and translational research in mind, we aimed to design shRNAs targeting *Gabarap* mRNA of both mouse and human origin. Given that the coding sequence (CDS) of *Gabarap* has only 354 nucleotides, it is noteworthy that there are only six regions where identical nucleotides span more than 19 base pairs, which is the minimal length required for shRNA design (43, 44). A total of five shRNAs that target the conserved sequence spanning both mouse and human *Gabarap* mRNAs were designed following shRNA design rules (43, 44) (Fig. 5*A*). To evaluate the knockdown efficiencies of these shRNAs, HEK293 cells that overexpressed mouse or human GABARAP were used. The expression of mouse GABARAP was effectively knocked down by each of the five shRNAs (Fig. 5 *B* and *C* and *SI Appendix*, Fig. S6 *A* and *B*). One shRNA, hereafter referred to as shRNA-1, was chosen for further study because of its better knockdown efficiency (Fig. 5 *B* and *C* and *SI Appendix*, Fig. S6 *A* and *B*). Next, western blot analysis revealed that the expression of both mouse and human GABARAP was knocked down in a dose-dependent manner by shRNA-1, but not by the control shRNA (Fig. 5 *D*–*G*). Notably, shRNA-1 specifically reduced the expression of GABARAP without affecting the expression of other Atg8 family proteins including GABARAPL1, GATE-16/GABARAPL2, and LC3β (Fig. 5 *F*–*I*).

### Reducing GABARAP Expression Does Not Affect Normal Hearing.

**Fig. 6.**
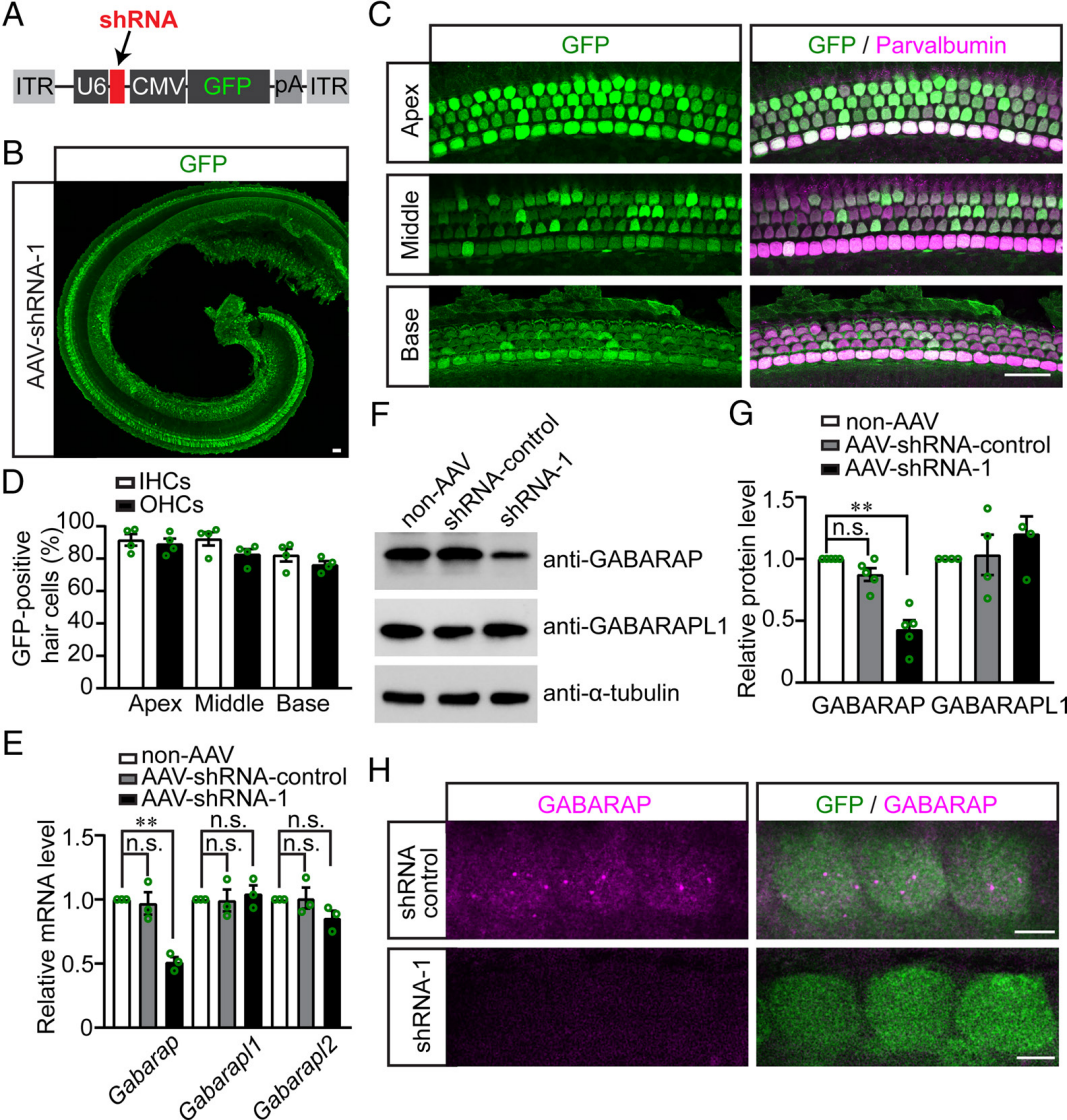
AAVs expressing shRNA-1 reduce the expression of GABARAP in hair cells. (*A*) AAV-shRNA vector used in this study. The expression of shRNA is driven by a U6 promoter and the expression of GFP is driven by a CMV promoter. (*B*) A representative low-magnification image of an injected cochlea showing the transduction level of AAV-shRNA-1. 2 µL of AAVs, expressing shRNA-1 and GFP, were injected into P1 mouse inner ear through the posterior semicircular canal. Cochleae were dissected at P7 and fixed for immunostaining. GFP (green) was used to identify the transduced cells. (*C*) Representative high-magnification images of the apical, middle, and basal regions of an injected cochlea. GFP (green) was used to identify the transduced cells, while Parvalbumin (magenta) was used to reveal the hair cells. Note, hair cells in the apical region showed a higher level of GFP expression. (*D*) Percentage of IHCs and OHCs in the apical, middle, and basal regions of cochlea transduced with AAV-shRNA-1. Note, more hair cells were transduced in the apical region of the cochlea compared with the basal region. Four mice were used for analysis. Data are represented as the mean ± SEM. (*E*) 2 µL of AAVs expressing either shRNA-1 or control shRNA were injected into the inner ears of P1 mice. Cochleae were dissected at P7, and mRNA was extracted. qRT-PCR was performed to evaluate the expression of *Gabarap*, *Gabarapl1,* and *Gabarapl2*. *Actb*, which encodes β-actin, was used as the endogenous control. Note, significantly reduced *Gabarap* expression in cochleae that were infected with AAV-shRNA-1. Three mice per group were used, and the experiment was repeated three times. Data are represented as the mean ± SEM. n.s., not significant, **P < 0.01 by Student’s *t* test. (*F*) 2 µL of AAVs expressing either shRNA-1 or control shRNA were injected into P1 mouse inner ear. Cochleae were dissected at P7 and lysed for western blot analysis. Note, reduced GABARAP expression in cochleae that were infected with AAV-shRNA-1. α-tubulin was used as the loading control. The experiment was repeated more than four times. (*G*) Quantification of the GABARAP and GABARAPL1 expression as shown in (*F*). Data are represented as the mean ± SEM. n.s., not significant, **P < 0.01 by Student’s *t* test. (*H*) 2 µL of AAVs expressing either shRNA-1 or control shRNA were injected into P1 mouse inner ear. Cochleae were dissected at P7 and fixed for immunostaining. GFP (green) was used to identify the transduced cells. Note, reduced GABARAP (magenta) expression in hair cells that were transduced with AAV-shRNA-1. [Scale bars, (*B* and *C*) 25 µm; (*H*) 5 µm.]

Next, an AAV expressing shRNA-1 under the control of a U6 promoter, hereafter referred to as AAV-shRNA-1, was produced and purified. AAV-shRNA-1 also expressed green fluorescence protein (GFP) under the control of a CMV promoter to identify the cells that were transduced (Fig. 6*A*). Wild-type mice were injected with AAV-shRNA-1 via the posterior semicircular canal at P1. Then, the cochleae were dissected at P7 and fixed for immunostaining. As indicated by the GFP fluorescence signal, ~92% of the IHCs and ~89% of the OHCs in the apical region, ~92% of the IHCs and ~82% of the OHCs in the middle region, and ~82% of the IHCs and ~76% of the OHCs in the basal region were transduced with AAVs (Fig. 6 *B*–*D* and *SI Appendix*, Fig. S6*C*). In addition to the higher AAV transduction rate at the apical region, most apical hair cells exhibited a stronger GFP fluorescence signal compared with basal hair cells (Fig. 6 *C* and *D*), which is in line with the previously reported tonotopic gradient of AAV transduction from the apex to the base in the inner ear (45).

Next, qRT-PCR, western blotting, and immunostaining revealed that the expression of GABARAP was significantly lower in hair cells after AAV-shRNA-1 administration than in those after control AAV-shRNA administration (Fig. 6 *E*–*H*). Treatment with AAV-shRNA-1 did not affect the expression of *Gabarapl1* or *Gabarapl2* in the inner ear (Fig. 6 *E*–*G*).

To determine whether reducing GABARAP expression through AAV-mediated RNAi affects normal hearing, the auditory functions of mice injected with AAV-shRNA-1 were characterized. The auditory brainstem responses to broadband click stimuli were not affected at 5 wk or 3 mo of age (*SI Appendix*, Fig. S7 *A, B, D*, and *E*). Furthermore, pure tone audiometry revealed that there was no significant change in hearing thresholds across the entire frequency spectrum, in the AAV-shRNA-1 treated mice compared with the untreated mice or control AAV-shRNA injected mice (*SI Appendix*, Fig. S7 *C* and *F*). These results indicate that AAV-shRNA-1, which reduces GABARAP expression, does not affect normal hearing.

### Reducing GABARAP Expression Prevents AG-Induced Hair Cell Death and Subsequent Hearing Loss.

**Fig. 7.**
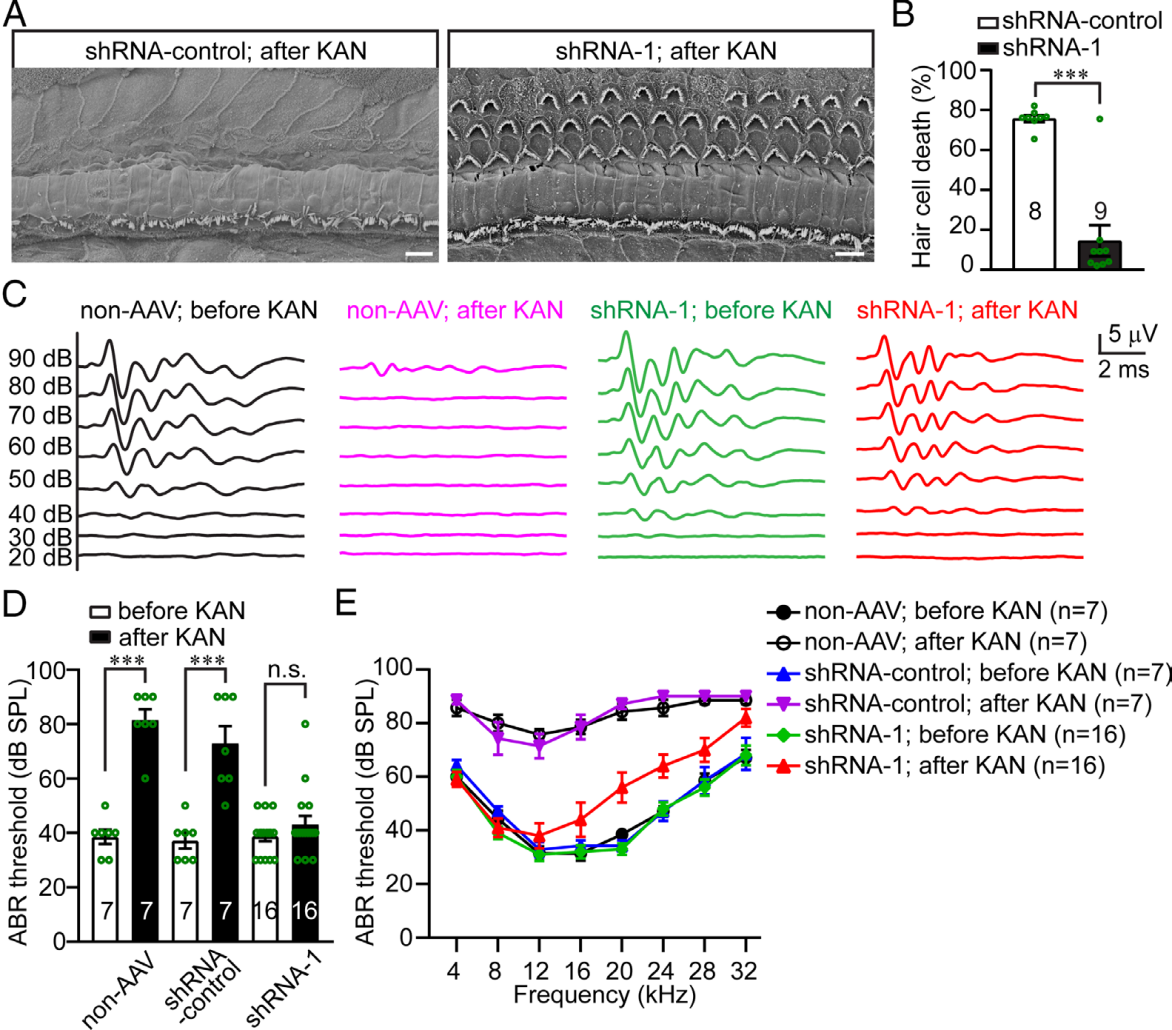
AAVs expressing shRNA-1 prevent AG-induced hair cell death and subsequent hearing loss. (*A*) 2 µL of AAVs, expressing shRNA-1, were injected into P1 wild-type mouse inner ear through the posterior semicircular canal. Mice at the age of 6 wk were treated with 800 mg/kg KAN twice daily for 14 consecutive days. Whole mounts from the middle part of the cochlea were analyzed by scanning electron microscopy. After KAN treatment, almost all the OHCs were lost in the mice that were not injected with AAV-shRNA-1. Limited hair cell death was observed in the mice that were injected with AAV-shRNA-1 after KAN treatment. (*B*) Percentage of hair cell death in AAV-shRNA-1 or AAV-shRNA-control injected mice after KAN treatment. The numbers of analyzed mice are indicated. Data are represented as the mean ± SEM. ***P < 0.001 by Student’s *t* test. (*C*) Representative click ABR traces from uninjected control mice and AAV-shRNA-1 injected mice, before and after 800 mg/kg KAN treatment. (*D*) ABR thresholds for click stimuli in uninjected control mice, and mice injected with AAV-shRNA-1 or AAV-shRNA-control, before and after 800 mg/kg KAN treatment. The numbers of analyzed mice are indicated. Data are represented as the mean ± SEM. n.s., not significant, ***P < 0.001 by Student’s *t* test. (*E*) ABR thresholds for pure tones in uninjected control mice, and mice injected with AAV-shRNA-1 or AAV-shRNA-control, before and after 800 mg/kg KAN treatment. The numbers of analyzed mice are indicated. Data are represented as the mean ± SEM. Significantly elevated hearing thresholds in uninjected control mice and AAV-shRNA-control injected mice after KAN treatment in all tested frequencies (*P* < 0.001 by Student’s *t* test). A mild but significant hearing threshold elevation in AAV-shRNA-1 injected mice after KAN treatment, at frequencies of 20, 24, 28, or 32 kHz (*P* < 0.001 by Student’s *t* test).

Next, we sought to determine whether AG ototoxicity can be prevented by specifically inhibiting GABARAP expression in the inner ear. Thus, 6-wk-old wild-type mice, which were injected with AAV-shRNA-1 via the posterior semicircular canal at P1, were given subcutaneous KAN injections at a dose of 800 mg/kg for 14 consecutive days. The cochleae were then dissected for scanning electron microscopy analysis. Mice without AAV injection or injected with control AAV-shRNA lost more than 75% of their hair cells after KAN treatment, whereas mice injected with AAV-shRNA-1 lost only ~17.8% of their hair cells (Fig. 7 *A* and *B*). These results suggest that reducing GABARAP expression in the inner ear prevents AG-induced hair cell death.

The auditory brainstem responses to broadband click stimuli were then measured. Treatment with 800 mg/kg KAN resulted in profound hearing loss in control mice that did not receive AAV injection or were injected with control AAV-shRNA. Remarkably, the same KAN treatment did not significantly affect the hearing thresholds of mice injected with AAV-shRNA-1 (Fig. 7 *C* and *D*). Furthermore, pure-tone audiometry revealed that KAN treatment induced profound hearing loss across the entire frequency spectrum tested (4 to 32 kHz) in control mice that did not receive AAV injection or were injected with control AAV-shRNA. In contrast, the same KAN treatment did not induce any significant hearing threshold elevation at lower frequencies (4, 8, 12, or 16 kHz) and only induced a ~15 dB hearing threshold elevation at higher frequencies (20, 24, 28, or 32 kHz) (Fig. 7*E*). The tonotopic gradient of AAV transduction, in which the infection rate is greater in apical hair cells responsible for detecting low-frequency sounds than in basal hair cells tuned to high-frequency sounds (Fig. 6 *B*–*D*), may provide a plausible explanation for the slightly different protective effects observed across different hearing frequencies. These findings suggest that reducing GABARAP in the inner ear prevents AG-induced hearing loss.

## Discussion

Annually, hundreds of millions of doses of AGs are consumed for the treatment of life-threatening Gram-negative bacterial infections; this causes an estimated 20 million cases of hearing loss every year (2). Therefore, it is imperative to investigate the mechanism by which AGs affect hair cell function in order to identify potential pharmacological targets, and subsequently to develop novel strategies to mitigate AG ototoxicity while preserving antimicrobial activity. We previously demonstrated that the RIPOR2-mediated autophagy pathway is essential for AG-induced hearing loss (13, 19). Genetic ablation in all tissues the expression of *Ripor2*, *Gabarap*, *Lc3β*, *Pink1*, or *Prkn* completely prevents AG-induced hair cell death and subsequent hearing loss (13, 19), paving the way for the rational design and subsequent testing of inhibitors that selectively target the proteins in this pathway. This study expands upon our previous work. Here, we report that *Gabarapl1* is another gene essential for AG ototoxicity. Furthermore, we demonstrated that hearing loss, induced by AG administered systemically, can be prevented by inhibiting *Gabarap* expression specifically in the inner ear. Additionally, by using an AAV-mediated gene knockdown approach, we successfully prevented hearing loss induced by a high dose of AG in wild-type mice.

Since AGs’ severe ototoxicity was first reported in the 1940s, significant efforts have been devoted to the development of pharmacological antidotes to mitigate this adverse effect. However, clinical trials have been discouraging. Most recently, large-scale drug screenings conducted on zebrafish and mouse cochlear explants have identified that ORC-13661, a high-affinity permeant blocker of the MET channel, inhibits the uptake of AGs by hair cells and prevents AG-induced high-frequency hearing loss (46). The safety and efficacy of ORC-13661 are being evaluated in a clinical trial approved by the FDA. As an alternative strategy, we aimed to mitigate AG ototoxicity by targeting the RIPOR2/GABARAP pathway. RIPOR2, an AG-binding protein previously identified in hair cells, is essential for AG-induced hearing loss, as is the RIPOR2/ GABARAP-mediated autophagy pathway (13, 19). AG-induced hearing loss can be completely or partially prevented by reducing the expression of *Ripor2*, *Gabarap*, *Lc3β*, *Pink1*, *Prkn* (13, 19), or *Gabarapl1*, as identified in this study. However, the autophagy pathway, as well as many of these central autophagy proteins, are not ideal pharmacological targets. Broadly targeting the autophagy pathway can lead to undesirable consequences because the survival of hair cells and auditory perception depend on several key autophagy proteins, such as Atg5 and Atg7 (47, 48). Genetic deletion of *Ripor2* or *Prkn* expression in mice results in profound or progressive hearing loss, respectively (13, 16, 17). Additionally, some familial forms of Parkinson's disease have been linked to loss-of-function mutations in *PINK1* or *PRKN* in humans, and genetic knockout of *Pink1* or *Prkn* expression in mice results in central nervous system defects (49–51). In this study, we identified *Gabarapl1* as another gene essential for AG-induced hearing loss. However, *Gabarapl1* is not an optimal pharmacological target either, as abolishing its expression can only partially prevent AG ototoxicity. Currently, *Gabarap* stands as one of the most promising targets for preventing AG ototoxicity. *Gabarap*-deficient mice are viable and exhibit no discernible morphological or behavioral abnormalities, as reported by Lexicon Genetics, Inc. (https://www.informatics.jax.org/knockout_mice/lexicon/407.html). Moreover, if RNAi entirely abolishes the expression of GABARAP, GABARAPL1 is most likely able to function redundantly and partially compensate for the absence of GABARAP, ensuring safety without the risk of overinhibition. Furthermore, we showed that knocking down *Gabarap* expression in the inner ear does not affect normal hearing. These findings suggest that *Gabarap* is one of the most promising therapeutic targets for preventing AG-induced hearing loss. However, further studies may uncover some unforeseen adverse effects resulting from inhibiting *Gabarap* expression. Thus, it is critical to identify additional proteins in the RIPOR2/GABARAP signaling pathway to provide additional targets for preventing AG-induced hearing loss.

Although both GABARAP and GABARAPL1 are abundantly expressed in cochlear hair cells, we found that GABARAP plays a more prominent role in AG ototoxicity (Fig. 4). qRT-PCR analysis (*SI Appendix*, Fig. S1*C*) revealed that *Gabarap* is expressed at significantly higher levels than *Gabarapl1* in the inner ear. Notably, previous RNA-seq studies, which provide more detailed gene expression profiles of the inner ear, show that *Gabarap* is more abundantly expressed in hair cells compared with *Gabarapl1* (20, 21). The higher mRNA expression level of *Gabarap* may explain its more prominent role in AG ototoxicity. Future protein-level analyses could provide deeper insights into this hypothesis. Given the lack of specific antibodies suitable for immunostaining, characterizing the protein expression levels of GABARAP and GABARAPL1 in cochlea using single-cell proteomics will be of particular interest. Another possibility is that, despite sharing 87% amino acid identity, GABARAP and GABARAPL1 may have slightly different functions in autophagy pathway and AG-induced hearing loss. Future biochemical screenings for their interaction partners, along with extensive characterization of *Gabarap^−/−^* and *Gabarapl1^−/−^* knockout mice, may provide further insights into the detailed functions of these proteins in AG-induced hearing loss.

The CDS regions of the mouse and human *Gabarap* mRNAs are highly conserved, whereas the 5′ and 3′ untranslated regions have very low similarities. Only a few shRNAs can be designed against sequence-identical regions between mouse and human, due to the short length of the *Gabarap* CDS. The evolutionarily conserved regions, which have been preserved through evolution, frequently encode functionally important amino acids and represent areas of lower redundancy in genetic sequences. Thus, targeting conserved regions may have higher efficacy while minimizing off-target side effects. Importantly, shRNAs that target identical sequences of mouse and human genes can be validated in mouse models in preclinical studies. Thus, although alternative shRNAs that target nonevolutionarily conserved sequences in the CDS regions or untranslated regions can be designed and tested, they are probably not ideal candidates.

In summary, we revealed that *Gabarapl1* is another essential gene for AG ototoxicity. By comparing *Gabarap* and *Gabarapl1* knockout mice, we demonstrated that *Gabarap* is a more promising therapeutic target for preventing AG ototoxicity. Significantly, AG ototoxicity can be effectively prevented by inhibiting *Gabarap* expression specifically in the inner ear. This demonstrates the feasibility of locally administering a compound to inhibit *Gabarap* expression, offering numerous advantages over systemic delivery, including fewer systemic adverse effects.

## Materials and Methods

### Animal Models.

*Gabarapl1^−/−^* mice were made using CRISPR/Cas9 technology on a C57BL/6J background. The CRISPR design tool (https://crispr.med.harvard.edu/sgRNAScorer/) was used to analyze the exon 2 sequence of *Gabarapl1*. Two sgRNAs, targeting genomic DNA sequence 5′-GGGTCCCTGATCTGGATAAGAGG-3′ and 5′-ATGGTTACCAGTGAGGTCGGAGG-3′, were synthesized by in vitro transcription following the published protocol (52) and microinjected into one-cell embryos. Genomic DNA from the offspring obtained by the embryo injections was then collected and screened by PCR using the following primers: 5′-GGAGTATTGGAAATGGGGACT-3′ and 5′-GCCAGCTCACTGAAGGAAAC-3′. The founder mice were then backcrossed with C57BL/6J mice for two generations. *Gabarap^−/−^* mice have been described previously (13). Both male and female mice were used in our experiments. We did not find any sex-based differences. More than three mice per group were used in each experiment.

All animal experiments were carried out in accordance with the NIH Guide and were approved by the Institutional Animal Care and Use Committee of Indiana University School of Medicine (IACUC Protocol 22069).

### Cell Culture, Transfection, and Western Blotting.

HEK293 (RRID:CVCL_0045) and HEK293T (RRID:CVCL_0063) cells were obtained from the American Type Culture Collection (ATCC) and maintained in DMEM (Life Technologies, 11965118) supplemented with 10% heat-inactivated fetal bovine serum (Fisher Scientific, MT35011CV) and 1% penicillin/streptomycin (Life Technologies, 15140122). Cells were grown at 37 °C in a 5% CO_2_ humidified atmosphere. HEK293 cells were transfected with plasmids expressing ATG8 proteins or shRNAs using Lipofectamine 2000 (Life Technologies, 11668019) following the manufacturer’s instruction. Three days after transfection, cells were collected and lysed using ice-cold RIPA buffer containing 50 mM HEPES pH 7.4, 150 mM NaCl, 1% Triton X-100 (MilliporeSigma, X100-500ML), and protease inhibitors (MilliporeSigma, 5056489001). Supernatants were collected after configuration at 16,000 x g at 4 °C for 15 min, loaded into SDS-PAGE gels and immunoblotted with appropriate antibodies. The intensity of the band was analyzed using ImageJ (NIH). The following antibodies were used for the experiments: anti-HA (Cell Signaling Technology, 3724, RRID:AB_1549585), anti-HA (MilliporeSigma, 11867423001, RRID:AB_390918), anti-GABARAPL1 (Proteintech, 11010-1-AP, RRID:AB_2294415), anti-GABARAP (abcepta, AP1821a, RRID:AB_2278762), anti-actin (Novus biologicals, NB600-501, RRID:AB_10077656), anti-α-tubulin (MilliporeSigma, T6199, RRID:AB_477583).

### Plasmids.

The coding sequence of *Gabarap* (NM_019749.4), *Gabarapl1* (NM_020590.4), *Gabarapl2* (NM_026693.5), *Lc3β* (NM_026160.4), *GABARAP* (NM_007278.1) or *GABARAPL1* (NM_031412.2) was inserted into the pEGFP-N3 vector (Clontech) between XhoI and NotI endonuclease restriction sites, replacing the EGFP coding sequence with that of the respective gene. The coding sequence of *Gabarapl1* (NM_020590.4) was cloned into the pAAV-Cbh vector (SignaGen). A translation start codon and an HA epitope tag (YPYDVPDYA; ATGTACCCATACGATGTTCCAGATTACGCT) was added at the N terminus of the above Atg8 genes. shRNA targeting *Gabarap* was inserted into AAV-shRNA plasmid (Addgene, 75438) (53) between BamHI and EcoRI restriction enzyme site. The sequences of *Gabarap* targeted by ShRNAs are as follows: shRNA-1: ACCATGAAGAAGACTTCTTTC; shRNA-2: TCTTTGTCAACAATGTCATTC; shRNA-3: CGGATAGGAGACCTGGACAAA; shRNA-4: ATGAAGTTCGTGTACAAAGAA; and shRNA-5: ATACCTGGTGCCTTCTGATCT.

### AAV Production and Injection.

AAVs were produced according to the published protocol (54). In brief, HEK293T cells, cultured in DMEM supplemented with 10% heat-inactivated fetal bovine serum, were transfected with pHelper, pAnc80L65AAP (Addgene, 92307) (26) and AAV expression plasmids in an equimolar ratio using polyethylenimine. One day after transfection, culture medium was changed to DMEM supplemented with 2% heat-inactivated fetal bovine serum. After 4 d of culture, cells were harvested and lysed. Supernatants were collected after configuration at 16,000×*g* at 4 °C for 15 min. Then, density gradient ultracentrifugation was performed by loading supernatant onto 60, 40, 25, and 15% iodixanol layers and centrifuge at 350,000×*g* for 1.5 h at 4 °C. To remove iodixanol in the AAV-containing fractions, buffer exchange was performed using 100 kD Amicon ultra-15 centrifugal filter unit (MilliporeSigma). AAV titer was determined by qRT-PCR using PowerUp™ SYBR™ Green Master Mix kit (Life Technologies, A25741) and QuantStudio™ 3 RT-PCR system (Life Technologies). AAVs (~ 1 × 10^10^ GC) were injected into P1 to P2 pups via posterior semicircular canal according to the published protocol (55).

### Cochlear Explant Culture and Immunostaining.

Cochlear explants were dissected and cultured according to our previously published protocol (13, 56). In brief, cochleae from P3 to P4 mice were dissected and cultured in DMEM/F12 medium (Life Technologies, 21041025) at 37 °C in a 5% CO_2_ humidified atmosphere. Then cochlear explants were treated with 1 mM GEN for 15 min at 37 °C, and fixed with 4% paraformaldehyde (PFA) in Hank’s Balanced Salt Solution (HBSS) (Life Technologies, 14175103) for 20 min. Samples were washed with HBSS three times and the tectorial membrane was then removed. Next, samples were blocked with 5% bovine serum albumin for 20 min at room temperature and incubated with primary antibodies overnight at 4 °C. After incubating with secondary antibodies for 2 h at room temperature, tissues were mounted in ProLong^®^ Antifade Reagents (Life technologies). Stacked images were then captured by using a fluorescence deconvolution microscope (Leica) or a confocal (Leica). The fluorescence intensity of RIPOR2 at the base of stereocilia was measured using ImageJ (NIH) as described previously (13). GTTR and FM1-43 (Life technologies) uptake assays were performed according to the published protocols (13, 57).

The primary antibodies employed in this study were as follows: anti-RIPOR2 (13), anti-HA (MilliporeSigma, 11867423001, RRID:AB_390918), anti-GABARAP (Provided by Drs. Regina Feederle and Andrew Flatley at Helmholtz Zentrum Munich, RRID: AB_2904599) (58), anti-GFP (Rockland Immunochemicals, 600-901-215, RRID:AB_1537402) and anti-parvalbumin (MilliporeSigma, SAB4200545, RRID:AB_2857970). Additional reagents included: Alexa Fluor 568-phalloidin (Life technologies, A12380), Alexa Fluor 488 goat anti-rabbit (Life technologies, A-11017), Alexa Fluor 488 goat anti-chicken (Life technologies, A-11039), Alexa Fluor 546 goat anti-rat (Life technologies, A-11081), Alexa Fluor 555 goat anti-mouse (Life technologies, A-21415) and Alexa Fluor 647 goat anti-mouse (Life technologies, A-21237).

### RNAscope In Situ Hybridization with Immunohistochemistry.

Inner ears from P5 wild-type C57BL/6J were dissected and fixed in 4% PFA in RNase-free Phosphate-Buffered Saline (Life Technologies) for 4 h. After cryoprotection in a 30% sucrose solution, samples were embedded in Tissue-Plus™ O.C.T. Compound (Fisher Scientific), sectioned using a Leica cryostat, and mounted on SuperFrost Plus slides (Fisher Scientific). RNAscope in situ hybridization was performed using the RNAscope® 2.5 HD Reagent Kit – RED (Advanced Cell Diagnostics, 322350) according to the manufacturer’s instructions and published protocols (59). Probes were designed and purchased from Advanced Cell Diagnostics. The following probes were used: *Gabarap* (Advanced Cell Diagnostics, 1232031-C1); *Gabarapl1* (Advanced Cell Diagnostics, 1295411-C1), *Myo7a* (Advanced Cell Diagnostics, 462771), and negative control probe- *DapB* (Advanced Cell Diagnostics, 310043). Following RNAscope in situ hybridization, samples were immunolabeled using an anti-MYO7A primary antibody (Proteus BioSciences, 25-6790) to visualize hair cells.

### KAN Treatment.

KAN treatment was performed as described (13). In brief, after dissolving KAN (Fisher Scientific, J17924) in USP grade saline (Fisher Scientific, Z1376), the concentration was adjusted to 160 mg/mL. Subcutaneous injections were administered twice daily for 14 consecutive days. There was a minimum of 8 h interval between the two injections within a day. The dosage of KAN was determined daily according to the body weight of the mice. Two to four days (Figs. 4 and 7) or 2 wk (*SI Appendix*, Fig. S5) after the final KAN injection, mice were subjected to morphological analysis and auditory function tests.

### qRT-PCR.

Cochleae were dissected from P7 pups. RNA was extracted using the RNeasy mini kit (Qiagen, 74104), and cDNA was reverse transcribed using Superscript III Reverse Transcriptase (Life Technologies). Then, qRT-PCR was performed using the QuantStudio™ 3 RT-PCR system (Life Technologies) and the PowerUp™ SYBR™ Green Master Mix kit (Life Technologies, A25741). Primers used to detect *Gabarap* were 5′- GCTCTGAGGGCGAGAAAATC-3′ and 5′-ACTGGTGGGTGGAATGACAT-3′; Primers used to detect *Gabarapl1* were 5′-GGACCACCCCTTCGAGTATC-3′ and 5′-GTTGTCCTCATACAGCTGGC-3′; Primers used to detect *Gabarapl2* were 5′-AGTCTCGGGCTCTCAGATTG-3′ and 5′-AGAGACACAAGCAGGAAGGG-3′. *Actb*, which encodes β-actin, was used as the endogenous control. Primers used to detect *Actb* were 5′-CATTGCTGACAGGATGCAGAAGG-3′ and 5′-TGCTGGAAGGTGGACAGTGAGG-3′.

### Scanning Electron Microscopy.

The experiment was performed as described (13, 16, 60). In brief, inner ears were dissected in fixative (0.05 mM HEPES Buffer pH 7.2; 2.5% glutaraldehyde; 4% PFA; 10 mM CaCl_2_; 5 mM MgCl_2_; 0.9% NaCl) and fixed for 1 h at room temperature. After dissecting the samples, the stria vascularis, Reissner's membrane, and tectorial membrane were removed. Following postfixation for 1 d at 4 °C in the same fixative, samples were washed with a washing buffer containing 0.05 mM HEPES Buffer (pH of 7.2) and 0.9% NaCl. Then, samples were fixed in 1% OsO_4_ for 1 h, serially dehydrated in ethanol, dried in a critical point drier (Autosamdri-815A, Tousimis), fine dissected, mounted on aluminum stubs, coated by gold, and imaged on a JEOL 7800F scanning electron microscope. To evaluate the survival of hair cells after KAN treatment, the percentage of hair cells that were lost in the middle turn of cochleae was quantified.

### ABR Measurement.

ABR experiments were performed as described previously (13, 16, 60), using TDT Bioacoustic system 3 and software (BioSig). In brief, mice were anesthetized using a cocktail of xylazine and ketamine. Three subdermal needle electrodes were placed at the vertex (active electrode), ipsilateral ear (reference electrode), and near the tail (ground electrode). The speaker was positioned 5 cm away from the mouse's ear. The sound stimulus started at an intensity of 90 dB and decreased stepwise to a subthreshold level. ABR thresholds were analyzed for the left ear and for a range of frequencies (for Pure Tone, 4 to 32 kHz). If no ABR wave was detected at maximum intensity stimulation, a nominal threshold of 90 dB was assigned.

### Statistics.

Each experimental group had a minimum of three animals of both genders from different litters. Precise numbers, sample size, repetitions, and statistic tests are indicated in the figures and figure legends. Data are represented as the mean ± SEM. Student's two-tailed unpaired *t* test and two-way ANOVA were used to determine statistical significance (n.s., not significant, *P < 0.05, **P < 0.01, ***P < 0.001).

## Supplementary Material

Appendix 01 (PDF)

## Data Availability

All study data are included in the article and/or *SI Appendix*.
