# Supplementary material for: Inhibition of GABARAP or GABARAPL1 prevents aminoglycoside- induced hearing loss
Source: Proc Natl Acad Sci U S A. 2025 Feb 10;122(7):e2416453122. doi: 10.1073/pnas.2416453122 (PMC11848329; doi:10.1073/pnas.2416453122)
Supplement: Supplementary file 1 — Appendix 01 (PDF) [file pnas.2416453122.sapp.pdf]

PNAS

Supporting Information for

Inhibition of GABARAP or GABARAPL1 prevents aminoglycoside-induced hearing loss

Jinan Li[1], Seung-Il Oh[1], Chang Liu[1] and Bo Zhao[1*]

[1]Department of Otolaryngology-Head and Neck Surgery, Indiana University School of Medicine, Indianapolis, IN 46202, USA.

[*]Correspondence to: Bo Zhao (zhaozb@iu.edu)

This PDF file includes:

Figures S1 to S7

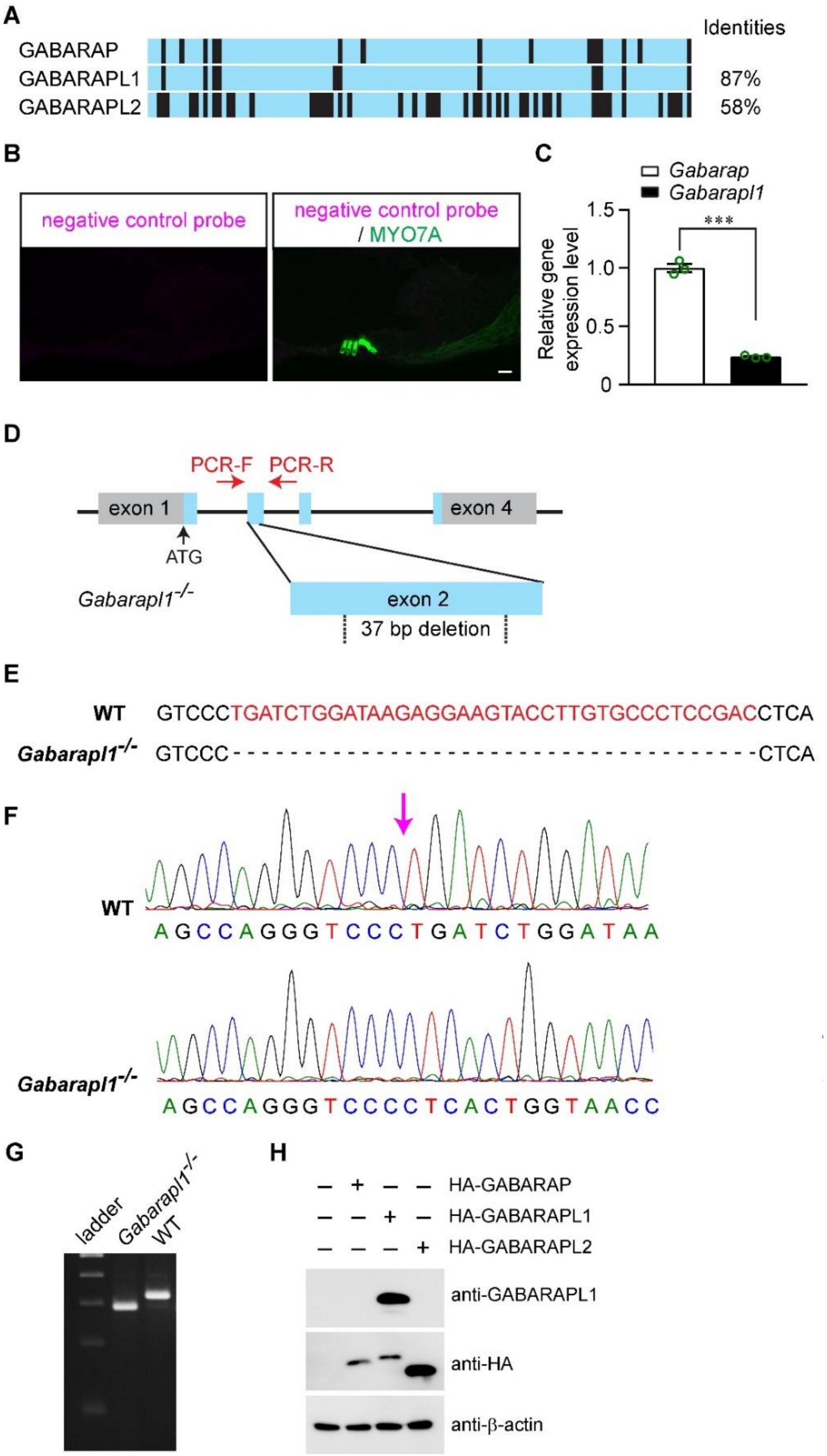

Figure S1. Generation of *Gabarapl1* null mutant mouse. (**A**) The alignment of the protein sequences of mouse GABARAP, GABARAPL1, and GATE-

16/GABARAPL2. Identical amino acids are colored cyan, while different amino acids are colored black. Note, GABARAP shares 87% amino acid identity with GABARAPL1, whereas it shares only 58% amino acid identity with GATE-16/GABARAPL2. (**B**) RNAscope *in situ* hybridization analyses were performed using a negative control probe-*DapB* (Advanced Cell Diagnostics, 310043) in P5 wild-type cochlea. Tissue sections were counterstained with MYO7A antibodies to visualize hair cells. Note, no RNAscope signal was detected. Scale bars: 20 μm. (**C**) Relative expression level of *Gabarap* and *Gabarapl1* in P7 cochlear epithelial cells. cDNA samples obtained from P7 wild-type cochleae were used as the template. Data are represented as the mean ± SE. ***$p < 0.001$ by Student's t test. (**D**) Diagram of the strategy to generate *Gabarapl1*-deficient mice. Two sgRNAs targeting exon 2 of *Gabarapl1* induced a 37-bp nucleotide deletion. Two primers, labelled as PCR-F and PCR-R, were designed for genotyping. (**E**) Genomic nucleotide sequences of the wild-type and *Gabarapl1*$^{-/-}$ mice. Note, the 37 bp deletion is highlighted in red. (**F**) Sanger sequence pattern of a PCR amplicon of the same region derived from wild-type and *Gabarapl1*$^{-/-}$ mice. The arrow points to the site of the 37 bp deletion. (**G**) Agarose gel electrophoresis of the PCR products amplified from the genomic DNA of wild-type and *Gabarapl1*$^{-/-}$ mice using primers shown in (D). (**H**) Plasmid expressing HA-tagged GABARAP, GABARAPL1, or GABARAPL2 was transfected into HEK293 cells. Western blotting was carried out to evaluate the specificity of the GABARAPL1 antibodies. Note, anti-GABARAPL1 antibodies specifically detected HA-GABARAPL1.

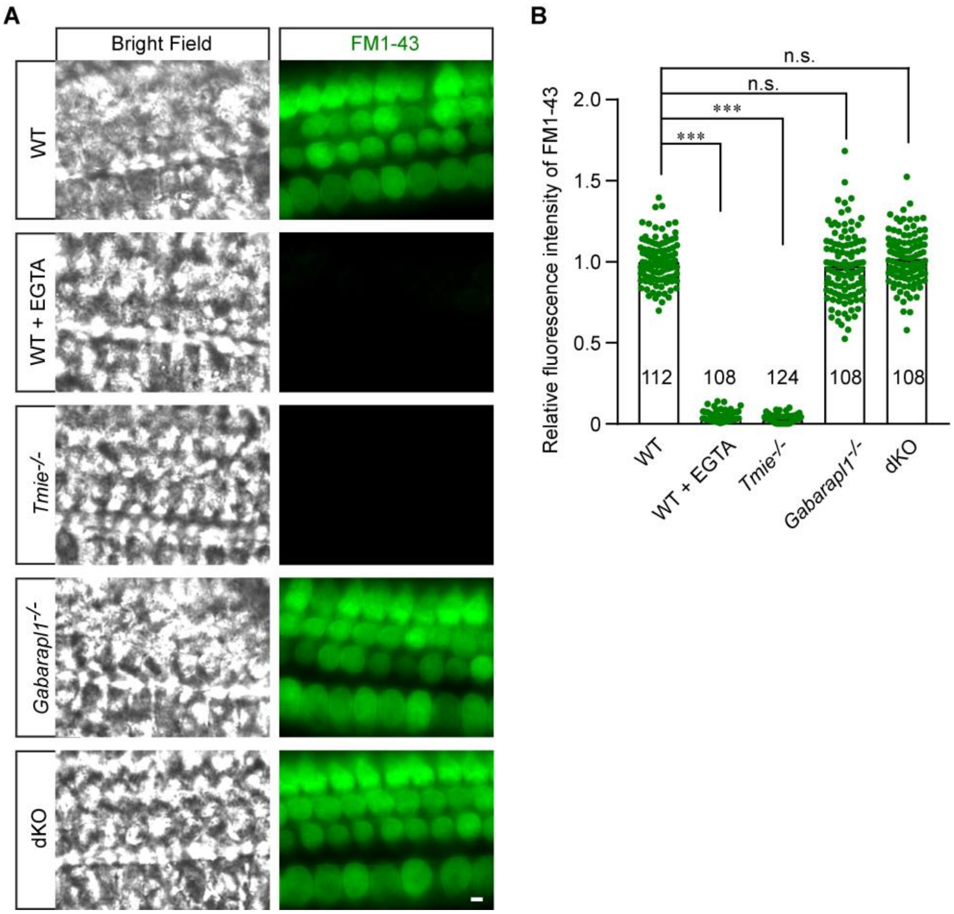

Figure S2. *Gabarapl1*$^{-/-}$ and *Gabarap*$^{-/-}$*Gabarapl1*$^{-/-}$ (dKO) hair cells show normal uptake of FM1-43 and GTTR. (**A**) P4 wild-type, *Tmie*$^{-/-}$, *Gabarapl1*$^{-/-}$, and dKO cochlear explants were incubated with 5 µM FM1-43 dye for 30 seconds at room temperature. Note, robust uptake of FM1-43 in wild-type, *Gabarapl1*$^{-/-}$, and dKO hair cells. Wild-type hair cells showed no rapid FM1-43 uptake after 30 min of pretreatment with 5 mM EGTA, nor did *Tmie*$^{-/-}$ hair cells. (**B**) Quantification results of the FM1-43 fluorescence intensity as shown in (A). In each group, three mice were used, and more than 30 hair cells per mouse were analyzed. The total number of cells analyzed per group is indicated in the figure. Data are represented as the mean ± SE. n.s., not significant, ***$p < 0.001$ by Student's t test. Scale bar: 5 µm.

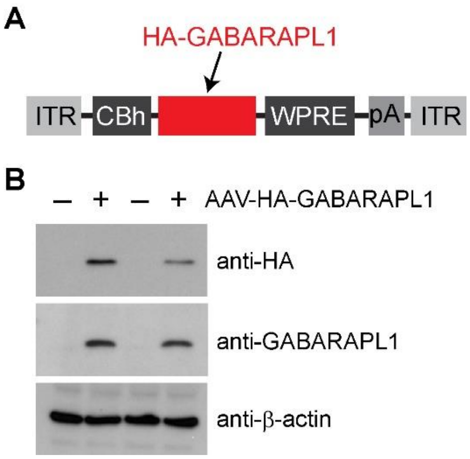

Figure S3. Expression of AAV-HA-GABARAPL1. (**A**) AAV-HA-GABARAPL1 vector used in this study contains a CBh promoter, HA-GABARAPL1 coding sequence, a WPRE element, a polyadenylation sequence (pA), and AAV inverted terminal repeats (ITRs). (**B**) AAVs, expressing HA-tagged GABARAPL1, were added to the culture medium of HEK293 cells. After three days of culture, HEK293 cells were collected and lysed using ice-cold RIPA buffer. Western blotting was carried out to evaluate the expression of HA-GABARAPL1. Both anti-HA and anti-GABARAPL1 antibodies detected a ~14 kDa specific band in samples infected with AAV-HA-GABARAPL1. β-actin was used as the loading control.

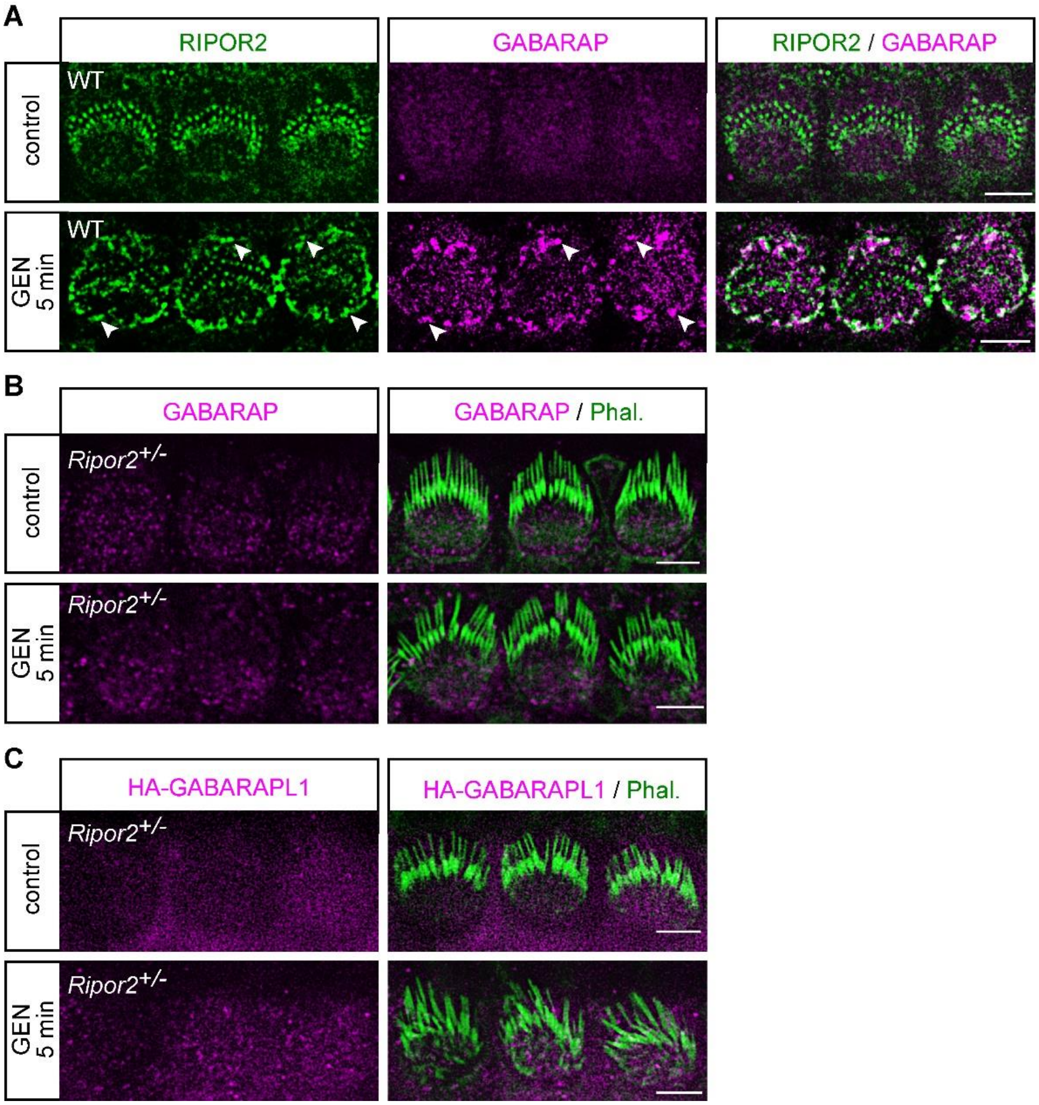

Figure S4. Translocation of GABARAP and GABARAPL1 in hair cells following AG exposure. (**A**) cochlear explants dissected from P4 wild-type mice were treated with 1 mM GEN and then stained for GABARAP and RIPOR2. Note, colocalization of GABARAP and RIPOR2 (arrowheads) in the pericuticular area following AG treatment. (**B**) P4 *Ripor2*$^{+/-}$ cochlear explants treated with 1 mM GEN exhibited minimal GABARAP accumulation in the pericuticular area. (**C**) AAVs expressing HA-GABARAPL1 were injected into the P1 *Ripor2*$^{+/-}$ inner ear via the posterior

semicircular canal. Cochlear explants were then dissected at P5 and exposed to GEN. Note, minimal HA-GABARAPL1 accumulation in the pericuticular area in *Ripor2*$^{+/-}$ hair cells. More than three mice were used per group in each experiment. Scale bars: 5 µm.

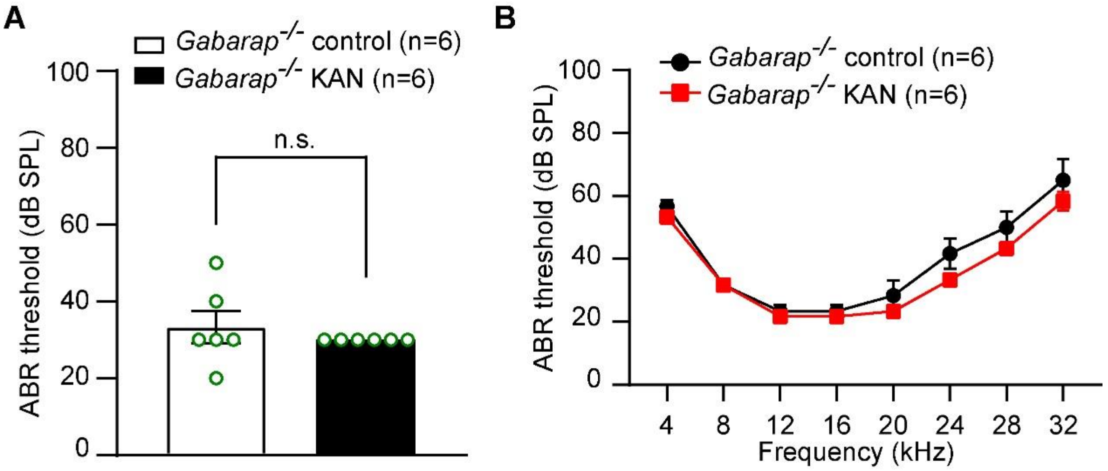

Figure S5. No hearing threshold elevation in *Gabarap*$^{-/-}$ mice following AG treatment. 3-week-old *Gabarap*$^{-/-}$ mice were treated with 800 mg/kg kanamycin (KAN) for 14 consecutive days. Two weeks after the final KAN injection, ABR thresholds were measured for click stimuli (A) and pure tones (B). The numbers of analyzed mice are indicated. Data are represented as the mean ± SE. n.s., not significant (Student's t test) in (A). In (B), two-way ANOVA test revealed no significant difference between non-treated and KAN-treated *Gabarap*$^{-/-}$ mice.

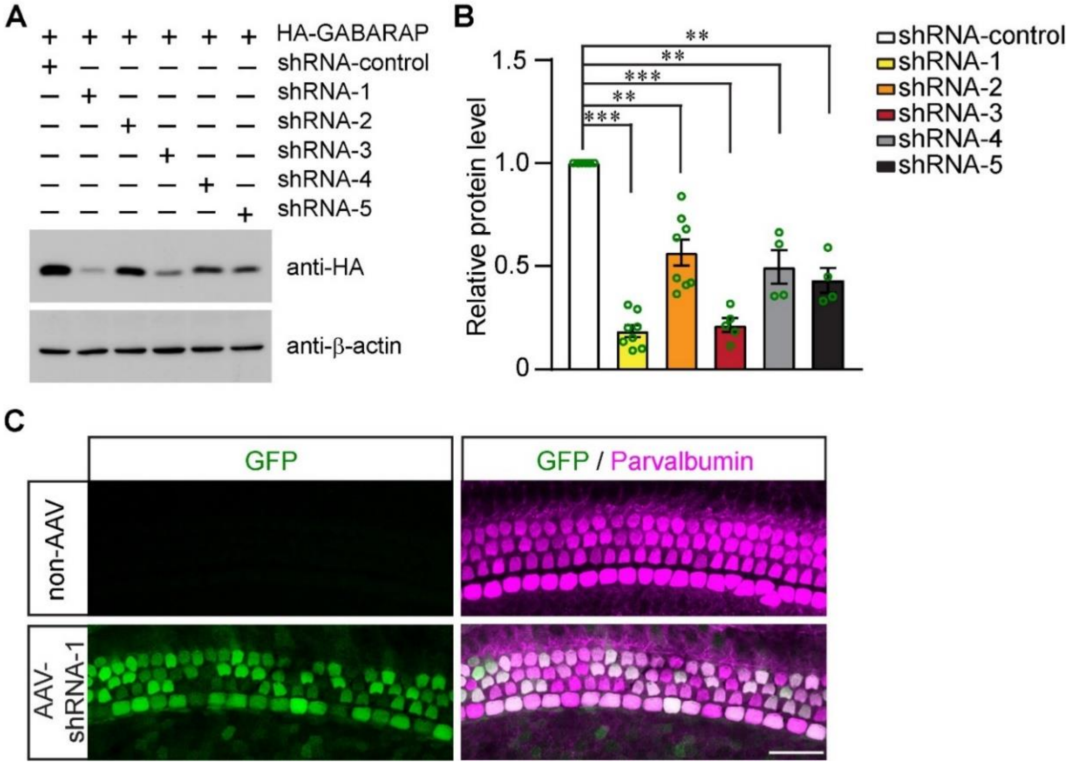

Figure S6. Validation of shRNAs that specifically target GABARAP. (**A**) Knockdown efficiencies of five shRNAs were tested. A plasmid expressing shRNA, along with a plasmid expressing HA-tagged mouse GABARAP, were transfected into HEK293 cells in an equimolar ratio. Western blotting was carried out to evaluate shRNA knockdown efficiency. Scrambled shRNA (shRNA-control) was used as the control, and β-actin was used as the loading control. (**B**) Relative expression level of HA-GABARAP as shown in (A). All values were normalized to β-actin and are shown as relative to samples treated with shRNA-control. Data are represented as the mean ± SE. $^{**}p < 0.01$, $^{***}p < 0.001$ by Student's t test. (**C**) Representative high-magnification images of the cochlea, with and without AAV transduction. GFP (green) was used to identify the transduced cells, while Parvalbumin (magenta) was used to reveal the hair cells. Note, there is no GFP signal in the cochlea in the absence of AAV transduction. Scale bar: 25 μm.

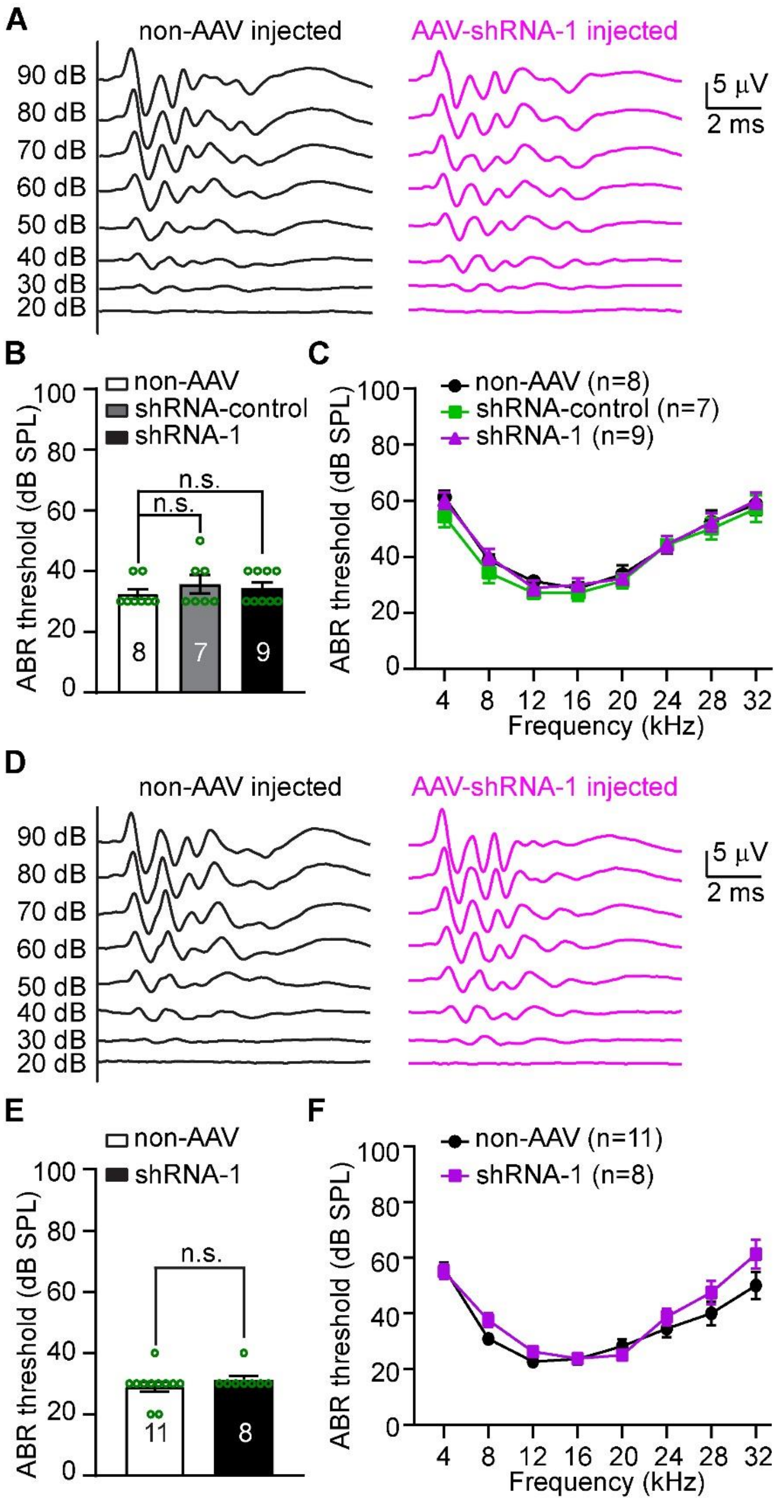

Figure S7. AAVs expressing shRNA-1 do not affect normal hearing. (**A**) 2 μl of AAVs, expressing shRNA-1 or control shRNA, were injected into P1 wild-type

mouse inner ear through the posterior semicircular canal. Auditory functions were characterized at the age of 5 weeks. Representative click ABR traces from 5-week-old uninjected control mice and AAV-shRNA-1 injected mice. (**B**) ABR thresholds for click stimuli in 5-week-old uninjected wild-type control mice, and mice injected with AAV-shRNA-1 or AAV-shRNA-control. The numbers of analyzed mice are indicated. Data are represented as the mean ± SE. n.s., not significant by Student's t test. (**C**) ABR thresholds for pure tones in 5-week-old uninjected control mice, and mice injected with AAV-shRNA-1 or AAV-shRNA-control. The numbers of analyzed mice are indicated. Data are represented as the mean ± SE. No significant difference by two-way ANOVA was detected between uninjected group and AAV-shRNA-1 injected group, or between AAV-shRNA-control injected group and AAV-shRNA-1 injected group. (**D**) Representative click ABR traces from 3-month-old uninjected control mice and AAV-shRNA-1 injected mice. (**E**) ABR thresholds for click stimuli in 3-month-old uninjected control mice and AAV-shRNA-1 injected mice. The numbers of analyzed mice are indicated. Data are represented as the mean ± SE. n.s., not significant by Student's t test. (**F**) ABR thresholds for pure tones in 3-month-old uninjected control mice and AAV-shRNA-1 injected mice. The numbers of analyzed mice are indicated. Data are represented as the mean ± SE. No significant difference by two-way ANOVA was detected between uninjected group and AAV-shRNA-1 injected group.
